# Methicillin-resistant *Staphylococcus pseudintermedius* synthesizes deoxyadenosine to cause persistent infection

**DOI:** 10.1080/21505594.2021.1903691

**Published:** 2021-03-29

**Authors:** Dorothea Bünsow, Eshraq Tantawy, Tjorven Ostermeier, Heike Bähre, Annette Garbe, Jesper Larsen, Volker Winstel

**Affiliations:** a Research Group Pathogenesis of Bacterial Infections; TWINCORE, Centre for Experimental and Clinical Infection Research, a Joint Venture between the Hannover Medical School and the Helmholtz Centre for Infection Research, Hannover, Germany; b Institute of Medical Microbiology and Hospital Epidemiology, Hannover Medical School, Hannover, Germany; c Research Core Unit Metabolomics, Hannover Medical School, Hannover, Germany; d Department of Bacteria, Parasites, and Fungi, Statens Serum Institut, Copenhagen, Denmark

**Keywords:** *Staphylococcus pseudintermedius*, nuclease, adenosine synthase A, deoxyadenosine, abscess

## Abstract

Methicillin-resistant *Staphylococcus pseudintermedius* (MRSP) is an emerging zoonotic pathogen of canine origin that causes an array of fatal diseases, including bacteremia and endocarditis. Despite large-scale genome sequencing projects have gained substantial insights into the genomic landscape of MRSP, current knowledge on virulence determinants that contribute to *S. pseudintermedius* pathogenesis during human or canine infection is very limited. Using a panel of genetically engineered MRSP variants and a mouse abscess model, we here identified the major secreted nuclease of *S. pseudintermedius* designated NucB and adenosine synthase A (AdsA) as two synergistically acting enzymes required for MRSP pathogenesis. Similar to *Staphylococcus aureus, S. pseudintermedius* requires nuclease secretion along with the activity of AdsA to degrade mammalian DNA for subsequent biosynthesis of cytotoxic deoxyadenosine. In this manner, *S. pseudintermedius* selectively kills macrophages during abscess formation thereby antagonizing crucial host immune cell responses. Ultimately, bioinformatics analyses revealed that NucB and AdsA are widespread in the global *S. pseudintermedius* population. Together, these data suggest that *S. pseudintermedius* deploys the canonical Nuc/AdsA pathway to persist during invasive disease and may aid in the development of new therapeutic strategies to combat infections caused by MRSP.

## Introduction

*Staphylococcus pseudintermedius* asymptomatically colonizes the skin, nares, and mucosal membranes of many wildlife and companion animals [1,2]. Carriage of *S. pseudintermedius* often reaches high frequencies in certain animal populations and represents a major risk factor for subsequent development of infections, particularly in canines with atopic dermatitis or other skin abnormalities [1–3]. In fact, this microbe is one of the most important bacterial pathogens of dogs and closely related species [1,2]. Specifically, *S. pseudintermedius* is a frequent cause of canine pyoderma, superficial skin and soft tissue infections, abscesses, ear and urinary tract infections, and invasive diseases such as bacteremia or endocarditis [2,4]. In addition, *S. pseudintermedius* represents an emerging cause of zoonotic infections in humans as this microbe can rapidly be transmitted via contact or bites from colonized or diseased animals to pet owners or veterinarian staff [2,5,6]. Clinical manifestations in humans encompass a similar spectrum as observed in dogs, including wound and soft tissue infections along with life-threatening diseases with fatal outcomes [2,5,7–10]. Although individuals at elevated risk often include patients with surgical procedures, bite wounds, medical implantation of foreign bodies or transplants, or cancer and immunosuppressive therapies, otherwise healthy individuals may also get colonized and acquire invasive disease, thus highlighting the zoonotic potential of *S. pseudintermedius* [5,11]. Combined with the global emergence of methicillin-resistant *S. pseudintermedius* (MRSP) and other multidrug-resistant variants, this pathogen has been recognized as an emerging threat to veterinary and human public health [2,12,13].

Current knowledge on pathogenicity and virulence determinants of *S. pseudintermedius* is very limited as strong genetic barriers interfere with available techniques for genetic manipulation. In particular, the presence of clustered regularly interspaced short palindromic repeats (CRISPR) and restriction modification systems have complicated basic biomedical research and the development of suitable genetic tools to manipulate *S. pseudintermedius* [14,15]. Nonetheless, genome analysis and a very limited number of studies revealed the presence of certain virulence factors that may contribute to *S. pseudintermedius* pathogenesis and immune evasion [2,16,17]. These factors include several toxins, proteases, staphylococcal protein A (SpA), and a secreted coagulase that promotes immune evasion [18–21]. Moreover, *S. pseudintermedius* produces several cell surface-displayed proteins, some of which contribute to attachment to host matrices and abscess formation [22,23]. For example, *S. pseudintermedius* surface protein L (SpsL) mediates binding to extracellular matrix proteins and affects development of abscesses during experimental skin infection [23,24]. Using recombinant protein and an *in vitro* approach, more recent work further demonstrated that *S. pseudintermedius* generates a cell surface-anchored 5ʹ-nucleotidase with homology to *Staphylococcus aureus* adenosine synthase A (AdsA) [25,26]. Both staphylococcal AdsA variants are capable of synthesizing immunomodulatory adenosine in blood [25,26]. Nevertheless, *S. aureus* AdsA has additional and crucial functions during abscess formation and persistent infection [27]. Specifically, *S. aureus* AdsA, together with a secreted nuclease (Nuc), converts neutrophil extracellular DNA traps (NETs) into deoxyadenosine (dAdo), which is cytotoxic for macrophages and other immune cells thereby supporting staphylococcal persistence in deep-seated abscess lesions and dissemination of disease [27–29]. However, it remains enigmatic whether *S. pseudintermedius* may also deploy the Nuc/AdsA signaling pathway along with biosynthesis of dAdo to promote abscess formation and invasive disease.

Here we identified the main secreted nuclease of *S. pseudintermedius* designated NucB that is capable to degrade DNA of various origin. Our results further suggest that NucB-mediated disruption of host DNA promotes the release of deoxyadenosine monophosphate (dAMP), which can be converted via *S. pseudintermedius* AdsA into cytotoxic dAdo. Ultimately, we show that production of dAdo by MRSP triggers immune cell death thereby supporting abscess formation in a mouse model of bloodstream infection. These insights may help to design new anti-infectives and preventive therapies to combat human or canine infections caused by multidrug-resistant *S. pseudintermedius*, including MRSP.

## Materials and methods

### Bacterial strains

All bacterial strains listed in Table S1 were grown in lysogeny broth (LB) or tryptic soy broth (TSB) at permissive temperatures. Media were supplemented with appropriate antibiotics (ampicillin 100 µg/ml; chloramphenicol 10 µg/ml).

### Cell culture

U937 cells were purchased from American Type Culture Collection (ATCC) and grown in Roswell Park Memorial Institute (RPMI) 1640 medium (Gibco) supplemented with 10% heat-inactivated fetal bovine serum (hi-FBS) according to the manufacturer’s instructions. RAW264.7 cells were obtained from ATCC and grown in Dulbecco’s Modified Eagle’s Medium (DMEM) supplemented with 10% hi-FBS according to the manufacturer’s instructions. Cells were grown at 37°C under 5% CO_2_. All mammalian cell lines used in this study are listed in Table S1.

### Molecular genetics

*S. pseudintermedius adsA, nucA, nucB*, or *nucC* were deleted by using pBASE6 and a published protocol [30]. In brief, the flanking regions of the appropriate genes were PCR-amplified from *S. pseudintermedius* genomic DNA and combined via overlap PCR using the primers listed in Table S2. Resulting PCR products were purified and cloned into linearized pBASE6 plasmid at the BglII and SacI restriction sites. Next, resulting knock-out plasmids were isolated from *E. coli* DC10B cells [31] and transferred to *S. pseudintermedius* via electroporation as described elsewhere [32]. Knock-out plasmids were integrated into the genome at a permissive temperature of 43°C and in the presence of 10 µg/ml chloramphenicol. Following integration, a counterselection step was performed at 30°C using anhydrotetracycline (0.2 µg/ml). Resulting clones were streaked onto tryptic soy agar (TSA) plates with or without chloramphenicol (10 µg/ml) and screened for plasmid loss. Chloramphenicol-sensitive colonies were screened via PCR and Sanger sequencing to confirm gene deletion. For complementation studies, the coding sequence of *S. pseudintermedius adsA* or *nucB* genes including its native promotor were PCR-amplified from *S. pseudintermedius* genomic DNA, purified, and cloned into the previously described shuttle plasmid pRB473 at the BamHI/SacI (*adsA*) or BamHI/PstI (*nucB*) restriction sites [33]. Resulting complementation plasmids pRB473-*adsA* or pRB473-*nucB* were transferred to *S. pseudintermedius* knock-out variants via electroporation as described before.

For protein overexpression studies, two new restrictions sites (SacI and BamHI) were added to pGEX-2T (GE Healthcare) via inverse PCR using the primers listed in Table S2. Next, primers pGEX-adsA-up and pGEX-adsA-dn were used to amplify a 1185-bp-long fragment of *adsA* starting after the signal peptide. Primers pGEX-nucB-up and pGEX-nucB-dn were used to amplify a 525-bp-long fragment of *nucB* starting after the signal peptide. The resulting PCR products were purified and cloned into the pGEX-2T plasmid as described above. The resulting plasmids were transferred to chemically competent *E. coli* BL21 cells for subsequent analysis. Construction of the pGEX-2T plasmid required for expression of *S. aureus* AdsA is described elsewhere [26].

### Protein purification

Recombinant and glutathione S-transferase (GST)-tagged variants of *S. aureus* AdsA (rSaAdsA), *S. pseudintermedius* AdsA (rSpAdsA), or *S. pseudintermedius* NucB (rSpNucB) were expressed in *E. coli* BL21 using pGEX-2T (GE Healthcare). Proteins were purified as described elsewhere using glutathione S-transferase affinity chromatography [26]. The N-terminal GST tag was removed via thrombin cleavage. Thrombin was removed from the samples by using benzamidine sepharose beads according to the manufacturer’s instructions (GE Healthcare). Purified proteins were analyzed by Coomassie-stained SDS-PAGE.

### Analysis of nuclease activity

To analyze nuclease production, the *S. pseudintermedius* strain panel was incubated overnight in TSB at 37°C. Two microliters of the resulting culture were spotted onto DNase test agar plates (Oxoid) and incubated overnight at 37°C. DNase test agar plates were flooded with 1 N HCl and visually analyzed for nuclease-mediated hydrolysis of DNA. Clear zones around bacterial colonies indicated nuclease production. Nuclease activity was further analyzed using a previously described agarose gel-based DNA degradation assay, with minor modifications [34]. In brief, 2 µl of filter-sterilized culture supernatants derived from the *S. pseudintermedius* panel or sterile TSB (negative control) were mixed with 2 µg host DNA and 40 µl nuclease buffer (300 mM Tris-HCl, pH 7.4; 3 mM MgCl_2_; 3 mM CaCl_2_) and incubated for 5 min at 37°C. All reactions were terminated using 12.5 µl of 0.33 M EDTA (pH 8.0), separated on a 1% agarose gel, and visualized with a UV transilluminator for image acquisition. A similar approach was used to test the activity of rSpNucB. Unless otherwise noted, 0.1 µg of rSpNucB was mixed with 2 µg of host DNA in nuclease buffer and incubated for 5 min at 37°C. Subsequently, reactions were stopped by using 12.5 µl of 0.33 M EDTA (pH 8.0). To analyze cation dependency of rSpNucB, reactions were also carried out in the presence of EDTA or EGTA or by using nuclease buffer that lacked CaCl_2_ or MgCl_2_. All samples were separated on agarose gels and analyzed as described above.

### Biochemical assays

AdsA activity and hydrolysis of dAMP were determined using a colorimetric phosphate detection kit (Abcam). Briefly, rSpAdsA (1.2 µg) or rSaAdsA (3.0 µg) were incubated with 2 µl of dAMP (5 mM) in reaction buffer (30 mM Tris-HCl, pH 7.5; 1.5 mM MgCl_2_; 1.5 mM MnCl_2_) for 16 h at 37°C. Controls lacked dAMP or recombinant AdsA. Following incubation, all reactions were terminated by adding EDTA (final conc. 50 mM) and analyzed for inorganic phosphate according to the phosphate detection kit manufacturer’s instructions. Alternatively, the *S. pseudintermedius* strain panel was grown in TSB to the early log phase and washed twice in reaction buffer (240 mM Tris-HCl, pH 7.4; 2.5 mM MnCl_2_; 2.5 mM MgCl_2_). Each strain (2.25 × 10^7^ CFU) was incubated with dAMP (final conc. 2.5 mM) in reaction buffer for 90 min at 37°C. Controls lacked bacteria or dAMP or included reaction buffer only. Following incubation, samples were centrifuged for 10 min at 16,000 × g. Hydrolysis of dAMP and associated release of inorganic phosphate in resulting culture supernatants was analyzed using the colorimetric phosphate detection kit as described above.

### Thin-layer chromatography

Formation of dAdo was analyzed via thin-layer chromatography (TLC) as described elsewhere with minor modifications [35]. Briefly, 2 µl of dAMP (5 mM) were mixed with 7.2 µg of rSpAdsA and incubated in reaction buffer (30 mM Tris-HCl, pH 7.5; 1.5 mM MgCl_2_; 1.5 mM MnCl_2_) for 16 h at 37°C. Controls lacked dAMP or rSpAdsA. Following incubation, all reactions were terminated by adding EDTA (final conc. 50 mM). Next, samples along with standards were applied to TLC plates (SIL G, Macherey-Nagel). TLC sheets were developed in the ascending direction at room temperature by using a water/isopropanol/ammonium bicarbonate mixture (25%: 75%: 0.2 M). The migratory positions of dAdo and dAMP were identified with pure dAdo and dAMP standards (Sigma) that were visualized under UV light at 254 nm.

Nucleoside formation using *S. pseudintermedius*-derived cell wall extracts was further analyzed using a previously described approach with minor modifications [26]. In brief, *S. pseudintermedius* overnight cultures were diluted 1:100 in TSB medium and grown to an optical density (600 nm) of 1.0. Bacteria were washed twice in sterile PBS, and 3 ml of the resulting suspension were centrifuged and suspended in 100 µl of TSM buffer (50 mM Tris-HCl, pH 7.5; 0.5 M sucrose; 10 mM MgCl_2_) containing lysostaphin (70 µg/ml). Reactions were incubated for 30 min at 37°C and centrifuged at 9,000 × g to obtain released cell surface proteins. Fifteen microliters of the resulting cell wall extracts were incubated with 3 µl dAMP (5 mM) for 30 min at 37°C. Samples were applied to TLC plates and analyzed as described above. Individual dAdo spots of independent biological replicates were also quantified by using the ImageJ processing package Fiji [36].

### LC-MS/MS-based analytics

To detect and quantify rSpAdsA-derived dAdo by LC-MS/MS, rSpAdsA was incubated with dAMP at 37°C as described above (see TLC section). Controls lacked dAMP or rSpAdsA. Following incubation, all reactions were terminated by adding EDTA (final conc. 50 mM). Subsequently, reversed phase chromatographic separation was performed using a Shimadzu HPLC-system (Shimadzu, Duisburg, Germany) consisting of two HPLC-Pumps (LC-30AD), a temperature controlled autosampler (SIL-30AC), a degasser (DGU-20A5), an oven (CTO-20AC), and a control unit (CBM-20A). A Hypercarb (30 × 4.6 mm; 5 µm; ThermoScientific, Waltham, Massachusetts, USA) connected to a C18 security guard (Phenomenex, Aschaffenburg, Germany) along with a 0.5 µm column saver were used. The column was maintained at 50°C. Solvent A was 10 mM ammonium acetate (pH 10; adjusted with 25% NH_3_). Solvent B was acetonitrile. For analyte separation, a gradient was applied: 0 to 8 min, 4 to 60% B; 8 to 12 min, 4% B. The flow rate was kept at 600 µL/min.

Analysis of target substances was carried out by a tandem mass spectrometer (5500QTRAP; ABSciex, Framingham, Massachusetts) equipped with an electrospray ionization source (ESI), operating in positive ionization mode and using an ion spray voltage of 4500 V. Further ESI parameters were: curtain gas (CUR): 30 psi, collision gas (CAD): 9, temperature: 600°C, gas 1: 60 psi and gas 2: 75 psi, respectively. For selected-reaction monitoring (SRM), the following mass transitions were used: dAMP: *m/z* 332 → 136 (quantifier), *m/z* 332→ 81 (identifier); dAdo: *m/z* 252 → 136 (quantifier), *m/z* 252 → 119 (identifier). The following mass transitions were used for the internal standards: tenofovir: *m/z* 288 → 176. Control of LC and the mass spectrometer as well as data sampling was performed by Analyst software (version 1.7, Sciex). For quantification, calibration curves were created by plotting peak area ratios of the target analyte and the internal standard versus the nominal concentration of the calibrators. The calibration curve was calculated using quadratic regression and 1/x weighing.

### Cytotoxicity assays

dAdo-mediated cytotoxicity was analyzed as described earlier [27–29]. Briefly, 4.0 × 10^5^ U937 cells per well were seeded in a 24-well plate and incubated for 48 h at 37°C under 5% CO_2_ in RPMI growth medium supplemented with 160 nM phorbol 12-myristate 13-acetate (PMA). Resulting U937-derived macrophages were washed and incubated in RPMI growth medium lacking PMA for additional 24 h. Alternatively, 2.0 × 10^5^ RAW264.7 cells per well were seeded in a 24-well plate and incubated for 24 h at 37°C under 5% CO_2_ in RAW264.7 growth medium. Next, wild-type *S. pseudintermedius* or mutant cells were grown overnight at 37°C in TSB, diluted 1:100 in RPMI 1640 medium, and grown at 37°C to 1.5 × 10^8^ CFU/ml. Subsequently, 1.8 × 10^8^ CFU were incubated in RPMI 1640 containing 28 μg/ml thymus DNA (Sigma) for 3 h at 37°C. Controls lacked thymus DNA or bacteria or included the *S. pseudintermedius nucB* or *adsA* mutant, which cannot generate dAdo. Following incubation, bacteria were removed by a brief centrifugation step. The resulting culture supernatants were filter-sterilized and incubated with U937- or RAW264.7-derived macrophages in the presence of 50 µM pentostatin (2′-deoxycoformycin [dCF]) for 18 h (U937) or 24 h (RAW264.7) as described before [27–29]. Following incubation, cells were detached using either trypsin-EDTA solution (U937-derived macrophages) or a cell scraper (RAW264.7-derived macrophages). Dead cells were stained with trypan blue and counted by using a microscope to calculate killing efficiency. To assess dAdo-mediated cytotoxicity using rSpAdsA, rSpAdsA was incubated with dAMP as described above (see TLC section). Controls lacked dAMP or rSpAdsA. The resulting reaction products were filter-sterilized and added to U937- or RAW264.7-derived macrophages in the presence of 50 µM dCF for 30 h. Following incubation, cells were detached and stained with trypan blue as described before.

### Ethics statement

All animal experiments were conducted in accordance with the local animal welfare regulations reviewed by the institutional review board and the Niedersächsisches Landesamt für Verbraucherschutz und Lebensmittelsicherheit (LAVES) under the permission number 33.19–42502-04-20/3528.

### Animal experiments

C57BL/6 mice were purchased from Janvier Laboratories and kept under specific pathogen-free conditions in our central mouse facility (TWINCORE, Center for Experimental and Clinical Infection Research, Hannover, Germany). For experiments, TSB overnight cultures of wild-type *S. pseudintermedius* or its *nucB* or *adsA* variants were diluted 1:100 in TSB and grown to an optical density (600 nm) of 0.5. Staphylococci were centrifuged (10 min, RT, 8,000 × g), washed twice in sterile PBS, and adjusted to 10^8^ CFU/ml. One hundred microliters of the bacterial suspension (10^7^ CFU) were administered intravenously (lateral tail vein) into 6- to 8-weeks-old female C57BL/6 mice. Five days post-infection, animals were killed. Livers and kidneys were dissected and examined for visible surface abscesses. Subsequently, organs were homogenized in sterile PBS containing 0.1% Triton X-100. Serial dilutions were prepared and plated onto TSA plates to enumerate staphylococci. For histopathology, dissected organs were fixed in 10% Formalin (Sigma), embedded into paraffin, and thin-sectioned. Thin sections of liver and renal tissues were stained with hematoxylin and eosin (H&E) and examined by microscopy according to standard laboratory protocols.

### Multilocus sequence typing (MLST)

MLST profiling was performed as described before [37,38]. All primers are listed in Table S2.

### Bioinformatics analysis

AdsA from *S. pseudintermedius* (GenBank accession no. ANS88668) and Nuc from *S. aureus* (NUC_STAAC, UniProtKB accession no. Q5HHM4) were used as queries in TBLASTN searches against a global collection of 622 *S. pseudintermedius* isolates [17]. SPAdes [39] was used to generate de novo assemblies for a subset of 364 isolates from the Sequence Read Archive (SRA) database, of which 49 failed to assemble due to corrupted sequence reads. Thus, the final dataset contained assemblies for 573 *S. pseudintermedius* isolates (Table S3). The MUSCLE algorithm [40] was used to construct multiple sequence alignments and calculate pairwise amino acid identities. Phylogenetic reconstruction was carried out with the maximum-likelihood program PhyML using default settings [41,42]. SignalP 5.0 [43] was used to predict the presence of signal peptides and the location of their cleavage sites.

### Statistical analysis

Statistical analysis was performed using GraphPad Prism (GraphPad Software, Inc., La Jolla, USA). Statistically significant differences were calculated by using statistical methods as indicated. *P* values <0.05 were considered significant.

## Results

### *Population genomics reveals the existence of multiple nuclease-encoding genes and* adsA *in* S. pseudintermedius *chromosomes*

**Figure 1.**
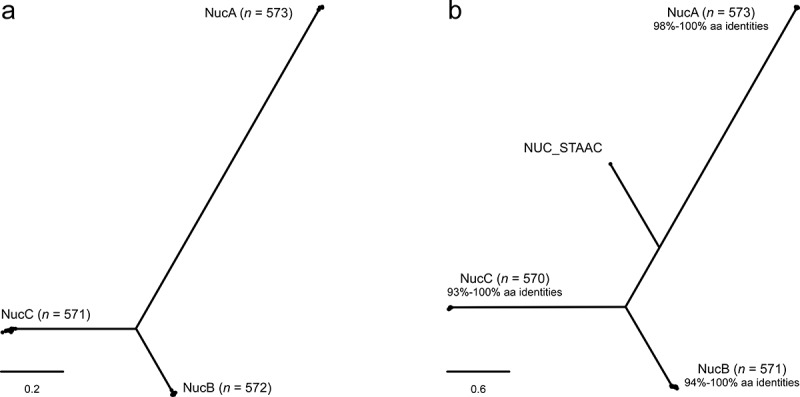
Maximum-likelihood phylogenies of Nuc proteins among 573 *S. pseudintermedius* isolates from the global collection. (a) TBLASTN hits (*n* = 1,716) using Nuc from *S. aureus* (NUC_STAAC) as query. (b) NUC_STAAC and TBLASTN hits (*n* = 1,714) using NucA, NucB, and NucC from *S. pseudintermedius* reference genome HKU10-03 as queries. The scale bars represent the number of amino acid (aa) substitutions per variable site. Phylogenetic clusters containing NucA, NucB, and NucC from *S. pseudintermedius* reference genome HKU10-03 are indicated. Two hits from the original TBLASTN searches with NUC_STAAC were not identified in the TBLASTN searches with NucA, NucB, and NucC due to the presence of premature stop codons, leading to a truncated NucB and NucC protein in *S. pseudintermedius* isolates SPSE-17-VL-OH-ON-0034 and 283,734,1391, respectively (data not shown)

To analyze whether *S. pseudintermedius* may utilize the Nuc/AdsA signaling pathway to persist during infection, we first scanned available *S. pseudintermedius* genome sequences for the presence of a nuclease- and AdsA-encoding gene. TBLASTN searches with Nuc from *S. aureus* (NUC_STAAC) identified 1,716 putative homologs among the 573 *S. pseudintermedius* isolates from a global collection (gathering threshold: identical sites > 30%, query coverage > 50%), which could be divided into three phylogenetic clusters (Figure 1(a)). Reciprocal TBLASTN searches with representative hits against the *S. pseudintermedius* reference genome HKU10-03 (GenBank accession no. NC_014925) showed that the hits mapped to three distinct ORFs, SPSINT_1053, SPSINT_1050, and SPSINT_0070, which were tentatively designated as *nucA, nucB*, and *nucC*, respectively, in accordance with the terminology used for *S. pseudintermedius* ED99 (GenBank accession no. CP002478). NucA (168 amino acids), NucB (213 amino acids), and NucC (276 amino acids) shared 36%, 47%, and 45% amino acid identities with NUC_STAAC (228 amino acids), respectively. Similar to NUC_STAAC, NucA and NucB contained a secretory signal peptide, suggesting that they are translocated across the plasma membrane via the general secretory pathway (Sec) and cleaved by signal peptidase I. In contrast, NucC did not contain any signal peptides. TBLASTN searches with NucA, NucB, and NucC identified homologs in 573, 571, and 570 of the 573 *S. pseudintermedius* isolates from the global collection (gathering threshold: identical sites > 90%, query coverage > 90%) (Figure 1(b) and Table S3). Two hits from the original TBLASTN searches with NUC_STAAC were not identified in the TBLASTN searches with NucA, NucB, and NucC due to the presence of premature stop codons, leading to a truncated NucB and NucC protein in *S. pseudintermedius* isolates SPSE-17-VL-OH-ON-0034 and 283,734,1391, respectively (data not shown). The three proteins were highly conserved (93%-100% amino acid identities) but only distantly related to each other (29%-41% amino acid identities). NUC_STAAC was most closely related to NucB (37%-39% amino acid identities), followed by NucC (32%-33% amino acid identities) and NucA (26%-28% amino acid identities) (Figure 1(b)). Lastly, AdsA was identified in 566 of the 573 *S. pseudintermedius* isolates from the global collection (gathering threshold: identical sites > 90%, query coverage > 90%) (Table S3). AdsA, including the 5ʹ-nucleotidase domain with the two signature sequences ILHTnDiHGrL and YdamaVGNHEFD [26,35,44], was highly conserved (96%-100% amino acid identities). These findings support that AdsA is widespread in the *S. pseudintermedius* population, as previously shown [25]. Thus, *S. pseudintermedius* isolates encode multiple nucleases and AdsA, thereby fulfilling all basic genomic requirements of the staphylococcal Nuc/AdsA pathway.

### S. pseudintermedius *secretes NucB to degrade host DNA of various origin*

**Figure 2.**
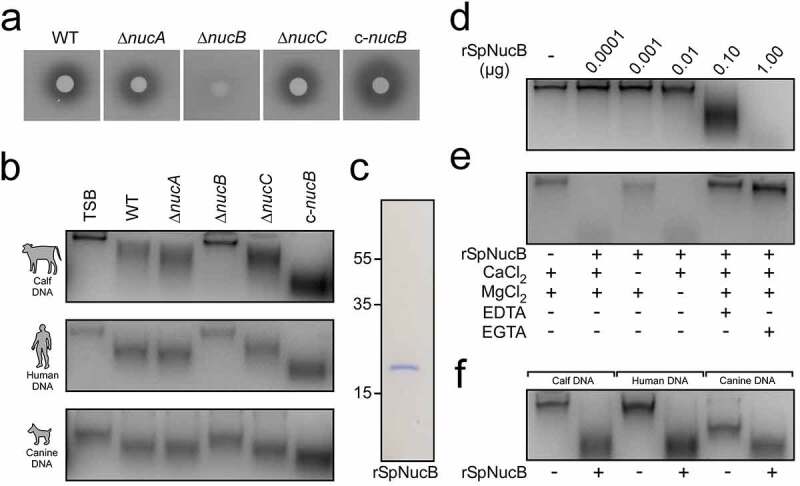
*S. pseudintermedius* secretes NucB to degrade host DNA. (a) Analysis of nuclease activity using DNase test agar plates. *S. pseudintermedius* overnight cultures were spotted onto DNase test agar plates, incubated overnight at 37°C, and flooded with 1 N HCl. Clear zones around bacterial colonies indicate nuclease production. (b) Detection of nuclease activity using an agarose gel-based DNA degradation assay. Filter-sterilized *S. pseudintermedius* culture supernatants or TSB were incubated at 37°C with calf, human, or canine DNA. Nuclease-mediated hydrolysis of host DNA was analyzed on agarose gels, which were visualized with a UV transilluminator for image acquisition. (c) SDS-PAGE analysis of purified rSpNucB. Numbers to the left of the SDS-PAGE indicate the migration of molecular weight markers in kilodaltons (kDa). (d-f) Activity and cation-dependency of rSpNucB in agarose gel-based DNA degradation assays. rSpNucB was incubated at 37°C with calf DNA or a variety of DNA substrates as indicated and analyzed as described above. Wild-type *S. pseudintermedius* DSM25713 (WT) or mutants lacking *nucA* (Δ*nucA*), *nucB* (Δ*nucB*), or *nucC* (Δ*nucC*) along with the complemented *nucB* mutant (c-*nucB*) are indicated. Representative images are shown

Since *S. pseudintermedius* seems to encode three putative nucleases, we wondered whether *S. pseudintermedius* may secrete one or more of these proteins for efficient degradation of host DNA. To identify the main secreted nuclease of *S. pseudintermedius*, we deleted the appropriate genes encoding for NucA, NucB, and NucC in a clinical MRSP isolate that was found to belong to the predominant MRSP ST71 lineage. The resulting *S. pseudintermedius* variants were validated via PCR-based genotyping and Sanger sequencing, and subsequently analyzed for nuclease activity by using commercially available DNase test plates (Figure 2(a) and Figure S1). Of note, this approach revealed that *nucB* deficiency in *S. pseudintermedius* completely abolished nuclease activity (Figure 2(a)). Deletion of *nucA* or *nucC* had no effect (Figure 2(a)). Similar results were obtained by using filter-sterilized culture supernatants of the mutant panel, which were incubated with calf thymus, human, or canine DNA and analyzed in an agarose-gel-based DNA degradation assay (Figure 2(b)). While *S. pseudintermedius* wild type, *nucA* or *nucC* mutant-derived culture supernatants efficiently degraded host DNA, *nucB* mutant-derived supernatants displayed strongly reduced nuclease activity, suggesting that NucB constitutes the main secreted nuclease of *S. pseudintermedius* (Figure 2(b)). To validate these results, we constructed a complementation plasmid (pRB473-*nucB*) that was transferred to the *S. pseudintermedius* ∆*nucB* strain via electroporation. This process completely restored the nuclease activity in both approaches, indicating that the observed phenotype of the *nucB* mutant is indeed caused by the absence of *nucB* expression (Figure 2(a,b)).

To explore the enzymatic activity of NucB in more detail, we expressed a soluble and affinity-tagged recombinant form of *S. pseudintermedius* NucB in *Escherichia coli* (hereafter termed rSpNucB), which was purified, released of its affinity tag, and subsequently used for enzymatic activity assays (Figure 2(c)). rSpNucB rapidly cleaved calf thymus DNA in a dose-dependent manner and required CaCl_2_ for full activity (Figure 2(d,e)). On the contrary, the addition of EDTA or EGTA, both known metal ion chelators, completely abolished the activity of rSpNucB (Figure 2(e)). Lastly, rSpNucB efficiently degraded various other DNA substrates including human or canine DNA (Figure 2(f)). Together, these experiments demonstrate that NucB represents the major secreted nuclease of *S. pseudintermedius* that facilitates degradation of host-derived DNA.

### S. pseudintermedius *deploys AdsA to synthesize cytotoxic deoxyadenosine*

**Figure 3.**
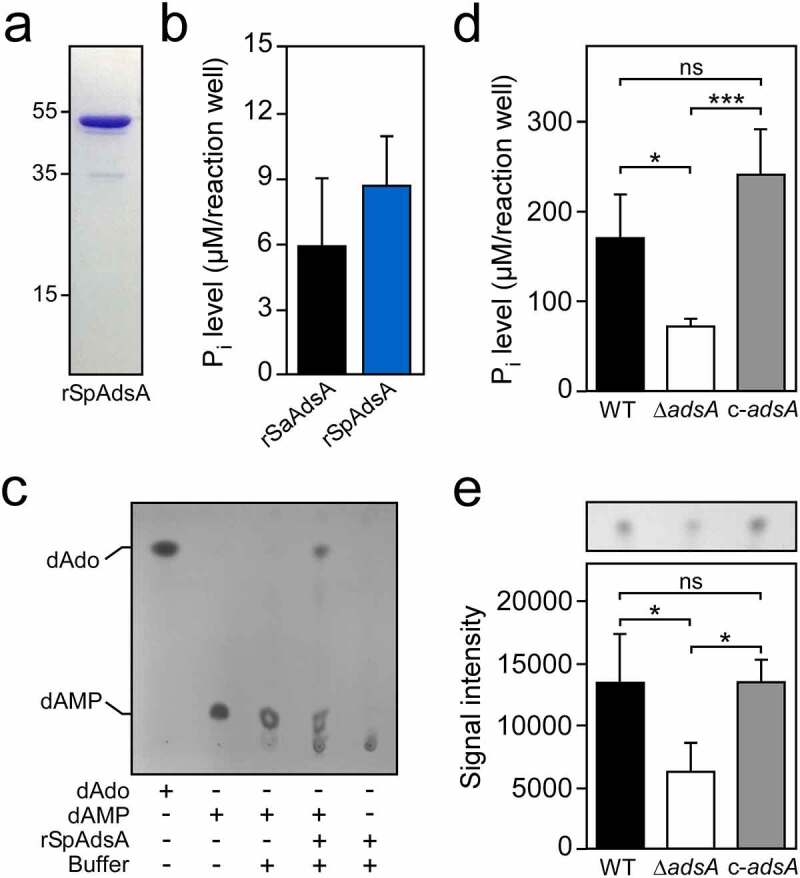
*S. pseudintermedius* utilizes AdsA to generate cytotoxic deoxyadenosine. (a) SDS-PAGE analysis of purified rSpAdsA. Numbers to the left of the SDS-PAGE indicate the migration of molecular weight markers in kilodaltons (kDa). (b) Hydrolysis of dAMP by recombinant *S. pseudintermedius* AdsA (rSpAdsA). Hydrolysis of dAMP was determined by assessing the release of inorganic phosphate (P_i_) after incubation of rSpAdsA with its substrate. Recombinant *S. aureus* AdsA (rSaAdsA) was used as a control. (c) Detection of dAdo formation by rSpAdsA. rSpAdsA was incubated with dAMP at 37°C. Controls lacking dAMP or rSpAdsA are indicated. Reaction products and the formation of dAdo were analyzed via TLC. The migratory positions of dAdo and dAMP were identified using pure dAdo and dAMP standards. (d) *S. pseudintermedius*-dependent hydrolysis of dAMP. The ability of *S. pseudintermedius* to hydrolyze dAMP in an AdsA-dependent manner was evaluated by assessing the release of inorganic phosphate (P_i_) using a malachite green-based colorimetric assay. (e) Analysis and quantification of *S. pseudintermedius*-dependent formation of dAdo. Lysostaphin-generated cell wall extracts of indicated strains were incubated with dAMP at 37°C and analyzed via TLC as described above. Individual dAdo spots of independent biological replicates were quantified by using the ImageJ processing package Fiji. Wild-type *S. pseudintermedius* DSM25713 (WT) or the mutant lacking *adsA* (Δ*adsA*) along with the complemented *adsA* mutant (c-*adsA*) are indicated. Representative images are shown. Data are the mean (± standard deviation [SD]) values from at least three independent determinations. Statistically significant differences were analyzed with one-way analysis of variance (ANOVA) and Tukey’s multiple-comparison test; ns, not significant (*P *> 0.05); *, *P *< 0.05; ***, *P *< 0.001

Earlier work demonstrated that *S. aureus* nuclease-mediated disruption of host DNA releases dAMP, which is converted via AdsA into cytotoxic dAdo [27]. Since *S. pseudintermedius* NucB also facilitates rapid cleavage of mammalian DNA resulting in the formation of free dAMP, we surmised that *S. pseudintermedius* might use a similar strategy and AdsA to synthesize dAdo during infection. To test this conjecture, we expressed a soluble and affinity-tagged recombinant form of *S. pseudintermedius* AdsA (hereafter termed rSpAdsA) in *E. coli* and purified the protein as described above (Figure 3(a)). Hydrolysis of dAMP was analyzed by using a commercially available phosphate detection kit, which measures the release of inorganic phosphate during the enzymatic reaction. Similar to *S. aureus* AdsA [27], rSpAdsA displayed potent nucleotidase activity and efficiently hydrolyzed dAMP (Figure 3(b)). Hydrolysis of dAMP by *S. pseudintermedius* AdsA and associated biosynthesis of dAdo was also assessed by using thin-layer chromatography (TLC). TLC-based analysis revealed that rSpAdsA catalyzed the conversion of dAMP to dAdo as dAdo exclusively accumulated in enzymatic reactions that contained the protein and dAMP (Figure 3(c)). These findings and the rSpAdsA-dependent biosynthesis of dAdo were further verified by HPLC-coupled tandem mass spectrometry (LC-MS/MS) (Figure S2). Together, these data suggest that *S. pseudintermedius* AdsA hydrolyzes dAMP for subsequent formation of dAdo.

To explore whether these results can also be recapitulated using live bacteria, we deleted the AdsA-encoding gene in MRSP strain DSM25713 and verified the mutation via PCR-based genotyping and Sanger sequencing (Figure S1). Next, wild-type *S. pseudintermedius* and the appropriate *adsA* mutant were incubated with dAMP and assayed for the release of inorganic phosphate using the malachite green-based colorimetric assay. Compared to wild-type bacteria, lack of *adsA* caused a significant reduction of detectable inorganic phosphate, suggesting that the *adsA* mutant lost the ability to hydrolyze dAMP (Figure 3(d)). Complementation by using a plasmid-borne copy of *adsA* restored the capacity of the *adsA*-deficient strain to hydrolyze dAMP to levels comparable to those seen in the parental strain, further indicating that *S. pseudintermedius* requires AdsA to synthesize dAdo (Figure 3(d)). To validate these results, we also digested peptidoglycan of the *S. pseudintermedius adsA* mutant panel with lysostaphin and incubated resulting cell wall extracts with dAMP for subsequent analysis via TLC. Cell wall extracts derived from the *adsA* mutant exhibited strongly reduced adenosine synthase activity, which could be restored to wild-type levels when the mutant was transformed with the complementation plasmid pRB473-*adsA* (Figure 3(e)). Image analysis and quantification of dAdo-specific signals following TLC confirmed further that the *adsA* mutant lost the capability to synthesize dAdo (Figure 3(e)). Collectively, these results demonstrate that *S. pseudintermedius* requires AdsA to hydrolyze dAMP, ultimately leading to the formation of cytotoxic dAdo.

### S. pseudintermedius *synthesizes dAdo to kill phagocytes*

**Figure 4.**
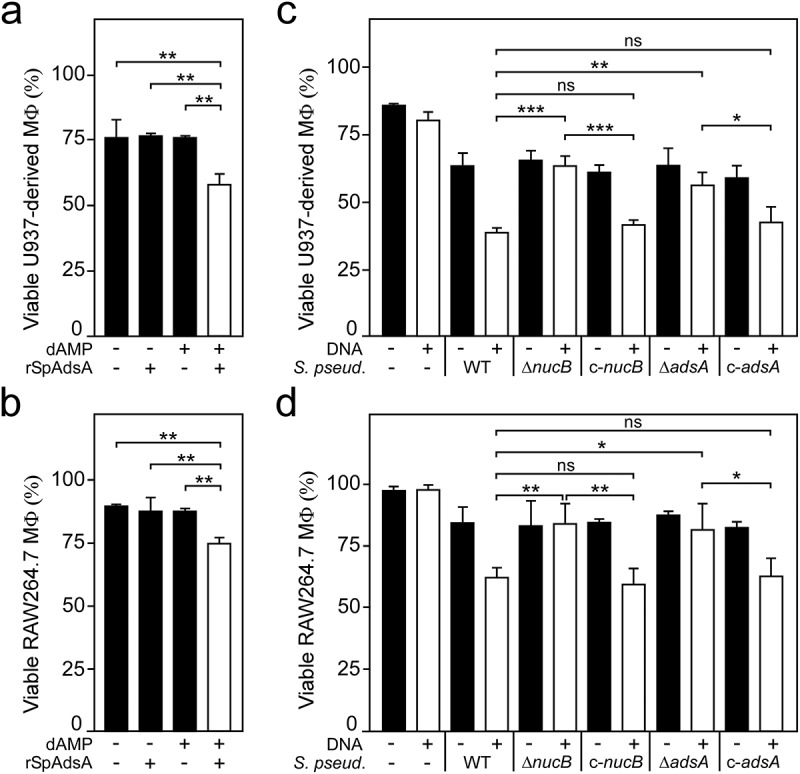
*S. pseudintermedius*-derived dAdo kills phagocytes. (a, b) Survival of U937- or RAW264.7-derived macrophages exposed to rSpAdsA-derived dAdo. rSpAdsA was incubated with dAMP, and reaction products containing dAdo were used to kill phagocytes. Controls included reaction buffer only or lacked rSpAdsA or dAMP as indicated with + and − symbols. (c, d) Survival of U937- or RAW264.7-derived macrophages following treatment with culture medium (RPMI) that had been conditioned by incubation with either wild-type *S. pseudintermedius* DSM25713 (WT) or mutants lacking *nucB* (Δ*nucB*) or *adsA* (Δ*adsA*) along with the complemented mutants (c-*nucB*; c-*adsA*) in the presence or absence of host DNA as indicated with the + and − symbols. All samples received adenosine deaminase inhibitor (50 μM dCF). Data are the mean (± standard deviation [SD]) values from three independent determinations. Statistically significant differences were analyzed with one-way analysis of variance (ANOVA) and Tukey’s multiple-comparison test; ns, not significant (*P *> 0.05); *, *P *< 0.05; **, *P *< 0.01; ***, *P *< 0.001

Previous studies revealed that *S. aureus*-derived dAdo exhibits strong toxigenic properties toward macrophages and other immune cells thereby promoting *S. aureus* pathogenesis during abscess formation [27,29]. Given that *S. pseudintermedius* AdsA can convert NucB-derived dAMP into dAdo, we wondered whether *S. pseudintermedius* might also utilize the Nuc/AdsA pathway and biosynthesis of dAdo to eliminate host phagocytes. To test this model, we incubated rSpAdsA with or without dAMP, and added resulting reaction products to human U937-derived macrophages. While enzymatic reactions that lacked rSpAdsA or dAMP did not cause cytotoxicity in this approach, rSpAdsA/dAMP-derived and dAdo-containing reaction products triggered cell death of human phagocytes (Figure 4(a)). Similar results were obtained for RAW264.7 mouse macrophages as only rSpAdsA/dAMP-derived and dAdo-containing reaction products, but not samples that lacked rSpAdsA or dAMP, promoted cell death (Figure 4(b)). Taken together, these experiments suggest that *S. pseudintermedius* AdsA-derived dAdo is capable to kill phagocytes of various origin.

To further investigate the role of *S. pseudintermedius*-derived dAdo in killing phagocytes, we took advantage of a previously described approach, and grew wild-type *S. pseudintermedius* or its *nucB* or *adsA* mutant variants that cannot synthesize dAdo in chemically defined medium supplemented with host DNA [27–29]. Following incubation, bacteria-free and conditioned culture media were filter-sterilized and added to U937- or RAW264.7-derived macrophages. As reported earlier for *S. aureus* [27–29], efficient killing of human or mouse phagocytes required *S. pseudintermedius* expressing *nucB* and *adsA* along with host DNA (Figure 4(c,d)). Specifically, addition of culture supernatants derived from wild-type *S. pseudintermedius* incubated with DNA triggered macrophage cell death, an effect that was significantly reduced when phagocytes were exposed to conditioned culture media obtained from *nucB* or *adsA* variants (Figure 4(c,d)). Since culture supernatants obtained from the complemented *nucB* or *adsA* mutants exhibited similar cytotoxic properties as those derived from the parental strain when incubated with DNA (Figure 4(c,d)), these experiments indicate that *S. pseudintermedius* utilizes dAdo signaling to eliminate host phagocytes in a manner requiring NucB and AdsA.

### MRSP requires NucB and AdsA for abscess formation

**Figure 5.**
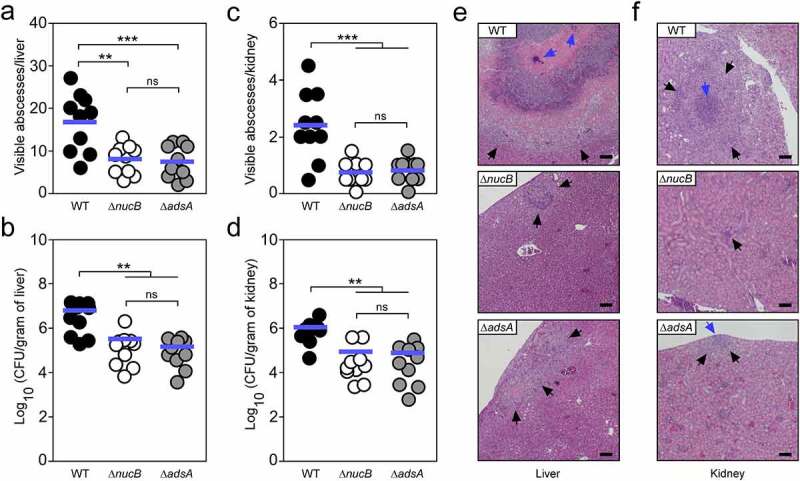
*S. pseudintermedius* requires NucB and AdsA for abscess formation. (a-d) Enumeration of visible surface abscesses and staphylococcal loads in livers and kidneys after intravenous injection of 10^7^ CFU of wild-type *S. pseudintermedius* DSM25713 (WT) or mutants lacking *nucB* (Δ*nucB*) or *adsA* (Δ*adsA*). Data for female C57BL/6 mice are displayed (*n* = 10). Bacterial burden was enumerated as log_10_ CFU per gram of tissue at 5 days post-infection. Horizontal blue bars represent the mean values of visible abscesses per organ (panels a and c) or indicate the mean CFU count in each cohort (panels b and d). Statistically significant differences were analyzed with one-way ANOVA and Tukey’s multiple-comparison test, or with the Kruskal–Wallis test corrected with Dunn’s multiple comparison if data did not pass normality distribution tests; ns, not significant (*P *> 0.05); **, *P *< 0.01; ***, *P *< 0.001. (e, f) Microscopic images of H&E–stained liver or renal tissues obtained after necropsy of female C57BL/6 mice infected with 10^7^ CFU of wild-type *S. pseudintermedius* DSM25713 or its *nucB* or *adsA* mutants. Arrows point to immune cell infiltrates (black) or replicating staphylococci (blue). Black bars depict a length of 100 μm. Representative images are shown

Finally, we wondered whether *S. pseudintermedius* requires the Nuc/AdsA pathway and associated biosynthesis of dAdo for invasive disease and abscess formation. To analyze this, female C57BL/6 mice were infected by intravenous inoculation of *S. pseudintermedius* strain DSM25713 or its *nucB* or *adsA* variants (10^7^ colony-forming units [CFU]). Five days post-infection, animals were killed and organs were removed for enumeration of visible abscess lesions and staphylococci in tissue homogenates. Wild-type *S. pseudintermedius* formed 16.7 (± 6.8 [SD]) abscesses per liver with a mean bacterial load of 6.6 × 10^6^ CFU per gram of tissue (Figure 5(a,b)). In contrast, abscess numbers and bacterial loads in livers were significantly reduced in animals infected with the *nucB* or *adsA* mutant (Figure 5(a,b)). Likewise, we observed fewer abscess lesions and a significant reduction in CFUs recovered from kidneys of *nucB* or *adsA* mutant-infected animals suggesting that NucB and AdsA are required for *S. pseudintermedius* abscess formation and pathogenesis (Figure 5(c,d)). In light of these findings, organs were also thin-sectioned and stained with hematoxylin and eosin (H&E) for subsequent histopathological analysis. Histopathological analysis of H&E-stained liver or renal tissues revealed that wild-type *S. pseudintermedius*-derived abscesses resemble, in principle, the structural organization of abscesses formed by *S. aureus* (Figure 5(e,f)). In particular, abscesses were clearly separated from healthy tissue and characterized by several foci of replicating staphylococci surrounded by a broad cuff of host immune cells (Figure 5(e,f)). Lesions obtained from *nucB* or *adsA* mutant-infected mice also displayed infiltrates of immune cells but appeared smaller in size and typically did not harbor a discernable organization of staphylococci, further demonstrating that *S. pseudintermedius* secretes NucB and AdsA to antagonize host immune cell responses via production of dAdo (Figure 5(e,f)). Collectively, these data indicate that NucB and AdsA significantly contribute to *S. pseudintermedius* virulence and abscess formation in a mouse model of infectious disease.

## Discussion

The global emergence and rapid dissemination of MRSP is a serious health threat in veterinary and human medicine [2,12,13]. Remarkably, MRSP and other multidrug-resistant clones of various sequencing types are spreading globally with steadily increasing incidence and prevalence rates in both healthcare and community settings [2,13,45,46]. Although large-scale genome sequencing projects and several epidemiological studies have gained new insights into the complexity of MRSP evolution and diversity, general understanding of *S. pseudintermedius* virulence and immune evasive attributes is still very limited. Specifically, *S. pseudintermedius*-derived factors that drive abscess formation or promote invasive diseases in the context of canine or human infections are largely unknown, highlighting the need of new research efforts to explore MRSP pathophysiology and infection dynamics.

Prompted by the fact that *S. pseudintermedius* genomes share certain features and virulence determinants with *S. aureus*, we here analyzed the impact of the staphylococcal Nuc/AdsA signaling pathway toward *S. pseudintermedius*-mediated abscess formation during invasive disease. Earlier work demonstrated that the combined activity of *S. aureus* Nuc and AdsA triggers formation of cytotoxic dAdo from NETs, which kills phagocytes by targeting the purine salvage pathway and non-inflammatory apoptosis [27–29]. In this manner, *S. aureus* escapes from NET-mediated killing and simultaneously excludes macrophages from infectious foci, thus promoting staphylococcal persistence and dissemination of disease [27,29]. Using a combination of biochemical approaches and a mouse abscess model, our results demonstrate that *S. pseudintermedius* utilizes a similar strategy to promote establishment of persistent lesions. Secreted NucB was required to dismantle host-derived DNA for subsequent biosynthesis of cytotoxic dAdo via AdsA, thereby boosting replication and survival of *S. pseudintermedius* in various mouse tissues. Given that neutrophils are less susceptible to apoptosis-inducing dAdo [27], and dAdo neither impairs the activity of neutrophils nor interferes with NET formation [47], we conclude that *S. pseudintermedius* has evolved NucB and AdsA for selective killing of macrophages. Nonetheless, differences in abscess formation and altered disease outcomes may also stem from additional mechanisms. In particular, the capacity of staphylococcal AdsA to hydrolyze pro-inflammatory purines such as ATP, ADP, or AMP may support *S. pseudintermedius* to subvert crucial immune surveillance pathways of the host [25,26]. Moreover, we note that NETs have antimicrobial and pathogen-immobilizing properties known to interfere with bacterial survival during infection [48]. NucB-mediated destruction of NETs along with the activity of AdsA may therefore not only lead to elevated levels of dAdo in infectious foci but also help *S. pseudintermedius* to prevent extracellular killing by activated neutrophils or other immune cells. Further, degradation of NETs could help *S. pseudintermedius* to seed new lesions in adjacent tissues or to enter circulating body fluids, which may result in abscess formation at new sites. In this regard, we note that canine pyoderma and superficial skin infections in dogs involve abscess formation and development of subcorneal pustules [49]. Likewise, abscesses and related pathological structures are the most common disease form caused by *S. pseudintermedius* in man [5,50]. Thus, the *S. pseudintermedius* NucB/AdsA signaling pathway along with dAdo biosynthesis and disruption of NETs may also affect the clinical outcome of acute and recurrent infections in humans underscoring the clinical significance. This may also hold true for other staphylococci belonging to the “*Staphylococcus intermedius* group” as some of these bacteria bear homologues of staphylococcal *adsA* and nuclease-encoding genes in their core genomes. Interestingly, some of these species (*i*.e., *Staphylococcus intermedius, Staphylococcus cornubiensis*, or *Staphylococcus delphini*) can cause abscesses, wound infections, or bacteremia in humans raising the possibility that these bacteria may also use a Nuc/AdsA signaling cascade to perturb host immune cell assemblies during pathogenesis [51–54]. Consequently, pharmacological inhibition or antibody-based targeting of Nuc/AdsA in combination with appropriate antibiotic treatment might become a suitable strategy to improve clinical outcomes of animal or human infections caused by MRSP and related pathogenic staphylococci that exploit the toxigenic properties of dAdo.

## Supplementary Material

Supplemental Material

## Data Availability

The datasets produced and/or analyzed during this study are available from the corresponding author on reasonable request.
