# Supplementary material for: Methicillin-resistant Staphylococcus pseudintermedius synthesizes deoxyadenosine to cause persistent infection
Source: Virulence. 2021 Mar 29;12(1):989–1002. doi: 10.1080/21505594.2021.1903691 (PMC8018352; doi:10.1080/21505594.2021.1903691)
Supplement: Supplemental Material [file KVIR_A_1903691_SM9123.pdf]

Supplemental Material

Title: Methicillin-resistant *Staphylococcus pseudintermedius* synthesizes deoxyadenosine to cause persistent infection

Authors: Dorothea Bünsow[a,b], Eshraq Tantawy[a,b], Tjorven Ostermeier[a,b], Heike Bähre[c], Annette Garbe[c], Jesper Larsen[d], Volker Winstel[a,b]#

[a] Research Group Pathogenesis of Bacterial Infections; TWINCORE, Centre for Experimental and Clinical Infection Research, a joint venture between the Hannover Medical School and the Helmholtz Centre for Infection Research, Hannover, Germany

[b] Institute of Medical Microbiology and Hospital Epidemiology, Hannover Medical School, Hannover, Germany

[c] Research Core Unit Metabolomics, Hannover Medical School, Hannover, Germany

[d] Department of Bacteria, Parasites, and Fungi, Statens Serum Institut, Copenhagen, Denmark

Address **correspondence** to Volker Winstel: winstel.volker@mh-hannover.de

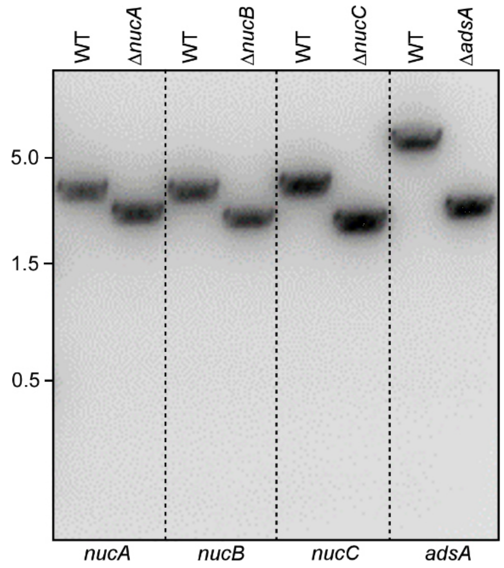

Figure S1. Confirmation of gene deletion in *S. pseudintermedius* DSM25713. Lack of *nucA* (Δ*nucA*), *nucB* (Δ*nucB*), *nucC* (Δ*nucC*), or *adsA* (Δ*adsA*) was confirmed by using a PCR-based genotyping strategy. PCR fragments and the absence of each gene was analyzed on agarose gels, which were visualized with a UV transilluminator for image acquisition. Wild-type (WT) *S. pseudintermedius* DSM25713-derived DNA and appropriate PCR products were used as controls. Numbers to the left of the agarose gel indicate the migration of DNA standards in kilobases (kb). Lack of each gene was also confirmed via Sanger sequencing (data not shown). A representative image is shown.

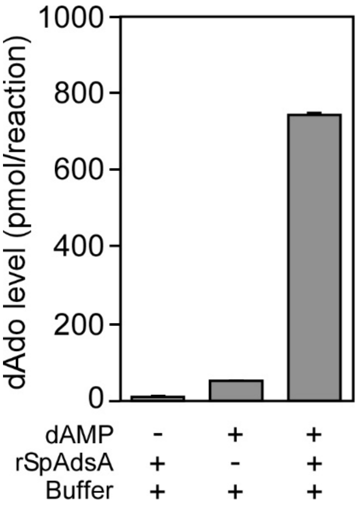

Figure S2. Detection and quantification of rSpAdsA-derived dAdo by LC-MS/MS. rSpAdsA was incubated with dAMP at 37°C. Controls lacking dAMP or rSpAdsA are indicated (+ and – symbols). Reaction products and the formation of dAdo were analyzed and quantified via LC-MS/MS. Data represent the mean (± standard deviation [SD]) values from two independent measurements.

Table S1. Bacterial strains and cell lines used in this study

**Bacterial strain**	**Descriptions**	**Reference**
E. coli DC10B	Δ*dcm* (DH10B background); Dam methylation only	[1]
E. coli DC10B pBASE6-*adsA*	DC10B bearing pBASE6-*adsA* knock-out plasmid	This study
E. coli DC10B pBASE6-*nucA*	DC10B bearing pBASE6-*nucA* knock-out plasmid	This study
E. coli DC10B pBASE6-*nucB*	DC10B bearing pBASE6-*nucB* knock-out plasmid	This study
E. coli DC10B pBASE6-*nucC*	DC10B bearing pBASE6-*nucC* knock-out plasmid	This study
E. coli DC10B pRB473-*adsA*	DC10B bearing pRB473-*adsA* complementation plasmid	This study
E. coli DC10B pRB473-*nucB*	DC10B bearing pRB473-*nucB* complementation plasmid	This study
E. coli BL21 (DE3) pGEX-2T-*adsA*	BL21 bearing pGEX-2T-*adsA* expression plasmid	This study
E. coli BL21 (DE3) pGEX-2T-*nucB*	BL21 bearing pGEX-2T-*nucB* expression plasmid	This study
S. pseudintermedius DSM25713	Clinical isolate, obtained from DSMZ strain collection	[2]
S. pseudintermedius Δ*adsA*	DSM25713 Δ*adsA*	This study
S. pseudintermedius Δ*adsA* pRB473-*adsA*	DSM25713 Δ*adsA* complemented with pRB473-*adsA*	This study
S. pseudintermedius Δ*nucA*	DSM25713 Δ*nucA*	This study
S. pseudintermedius Δ*nucB*	DSM25713 Δ*nucB*	This study
S. pseudintermedius Δ*nucC*	DSM25713 Δ*nucC*	This study
S. pseudintermedius Δ*nucB* pRB473-*nucB*	DSM25713 Δ*nucB* complemented with pRB473-*nucB*	This study
U937	U937 cell line, ATCC® CRL-1593.2™	ATCC
RAW264.7	RAW264.7 cell line, ATCC® TIB-71™	ATCC

Table S2. Oligonucleotides used in this study

Primer	Sequence	Application	Reference
F1-adsA-up	ATCGAGCTCAATCAAAATGTGTACCTCTGAATATTGTTAGG	Generation of pBASE6-*adsA*	This study
F1-adsA-dn	ATAAAACCTCCTTATTGTCATTACAATTTTATCCT	Generation of pBASE6-*adsA*	This study
F2-adsA-up	TGACAATAAGGAGGTTTTATATGAGTATCCCCCCTAAAGTTGAGTGGA	Generation of pBASE6-*adsA*	This study
F2-adsA-dn	GAGAAGATCTGTCAAATATTGATTTTGATAGTAGAGCGCTTATACT	Generation of pBASE6-*adsA*	This study
F1-nucA-up	ATCGAGCTCATT TTGTATACAAGGTAGGAGGTATCTATACAC	Generation of pBASE6-*nucA*	This study
F1-nucA-dn	AGCGACTCCTTTTTATGTATGATTAACACC	Generation of pBASE6-*nucA*	This study
F2-nucA-up	ACATAAAAAGGAGTCGCTAACATTAAAGACACTTTGAGGCTAATATACAG	Generation of pBASE6-*nucA*	This study
F2-nucA-dn	GAGAAGATCTAACGAGCCCCCTATCAAAATGCACAAAAT	Generation of pBASE6-*nucA*	This study
F1-nucB-up	ATCGAGCTCATCATCGTGATTGTTGCAGTGATAATCGGT	Generation of pBASE6-*nucB*	This study
F1-nucB-dn	GCATTTTAGCCTCCTTAAAATGTCCATTTTAT	Generation of pBASE6-*nucB*	This study
F2-nucB-up	TTAAGGAGGCTAAAATGCGTAGTAACAGAAAAAAATAACAGAAGCAGTAC	Generation of pBASE6-*nucB*	This study
F2-nucB-dn	GAGAAGATCTCGCTGTTGAACGATTTAAAGGCACATGTT	Generation of pBASE6-*nucB*	This study
F1-nucC-up	ATCGAGCTCATTCTGCTCATTATGATGACACATTTCGTT	Generation of pBASE6-*nucC*	This study
F1-nucC-dn	TCCCCCAACTATAACAAATAGAATAATAAGAG	Generation of pBASE6-*nucC*	This study
F2-nucC-up	TATTTGTTATAGTTGGGGGAAAATGTATAAATAACAGGCTAGAAGCTGTC	Generation of pBASE6-*nucC*	This study
F2-nucC-dn	GAGAAGATCTTAGCTAGAAAACCATAGATAACGCTAGCT	Generation of pBASE6-*nucC*	This study
473-adsA-up	ATCGGATCCATGGGCATACTCGACTCCTTTGTTTGGAAA	Generation of pRB473-*adsA*	This study
473-adsA-dn	GAGAGAGCTCTTATGCGTGGTGCTTTTGACGTCGCATAAT	Generation of pRB473-*adsA*	This study
473-nucB-up	ATCCTGCAGATTAAATCCAAGAGAGAACGGCGTAATTCC	Generation of pRB473-*nucB*	This study
473-nucB-dn	GAGAGGATCCTTATGACGCCAGACTTCCAATATTTGCTGT	Generation of pRB473-*nucB*	This study
pGEX-BamHI	GAGAGGATCCACGCGGAACCAGATCCGATTTT	Amplification of pGEX-2T	This study
pGEX-SacI	ATCGAGCTCATCGTGACTGACTGACGATCTGCCTCG	Amplification of pGEX-2T	This study
pGEX-adsA-up	ATCGGATCCGCCGAACAAACATCACAACAAGCAGCT	Generation of pGEX-2T-*adsA*	This study
pGEX-adsA-dn	GAGAGAGCTCTTATGCTTCATTCGCTTTATCGACTTGTGCTTT	Generation of pGEX-2T-*adsA*	This study
pGEX-nucB-up	ATCGGATCCGCTACGGGAAAATCTACCGTCACATCAT	Generation of pGEX-2T-*nucB*	This study
pGEX-nucB-dn	GAGAGAGCTCTTATGACGCCAGACTTCCAATATTTGCTGT	Generation of pGEX-2T-*nucB*	This study
tuf-forward	CAATGCCACAAACTCG	MLST typing	[3]
tuf-reverse	GCTTCAGCGTAGTCTA	MLST typing	[3]
cpn60-forward	GCGACTGTACTTGCACAAGCA	MLST typing	[3]
cpn60-reverse	AACTGCAACCGCTGTAAATG	MLST typing	[3]
pta-forward	GTGCGTATCGTATTACCAGAAGG	MLST typing	[3]
pta-reverse	GCAGAACCTTTTGTTGAGAAGC	MLST typing	[3]
purA-forward	GATTACTTCCAAGGTATGTTT	MLST typing	[4]
purA-reverse	TCGATAGAGTTAATAGATAAGTC	MLST typing	[4]
fdh-forward	TGCGATAACAGGATGTGCTT	MLST typing	[4]
fdh-reverse	CTTCTCATGATTCACCGGC	MLST typing	[4]
ack-forward	CACCACTTCACAACCCAGCAAACT	MLST typing	[4]
ack-reverse	AACCTTCTAATACACGCGCACGCA	MLST typing	[4]
sar-forward	GGATTTAGTCCAGTTCAAAATTT	MLST typing	[4]
sar-reverse	GAACCATTCGCCCCATGAA	MLST typing	[4]

Table S3. Identification of nuclease- and AdsA-encoding genes in the global collection of 622 *Staphylococcus pseudintermedius* isolates

Isolate name	Database	Sequence accession no.	MLST	Assembly	AdsA	NucA	NucB	NucC
283734,1457	PATRIC	283734,1457	17	1	1	1	1	1
ED99	RefSeq	NC_017568	25	1	1	1	1	1
17-34405	SRA	SRR10076055	45	0	ND	ND	ND	ND
17-B24281	SRA	SRR10076059	45	0	ND	ND	ND	ND
NYVetLIRN-31	SRA	SRR5875113	45	1	1	1	1	1
SPSE-18-VL-SD-MI-0030	SRA	SRR8815933	45	1	1	1	1	1
SPSE-18-VL-SD-NC-0025	SRA	SRR8815951	45	1	1	1	1	1
SL/076	RefSeq	MQMP00000000	45	1	1	1	1	1
SL/085	RefSeq	MQNB00000000	45	1	1	1	1	1
SL/094	RefSeq	MQNC00000000	45	1	1	1	1	1
SL/114	RefSeq	MQND00000000	45	1	1	1	1	1
VTH737	RefSeq	QGOX00000000	45	1	1	1	1	1
283734,1413	PATRIC	283734,1413	45	1	1	1	1	1
742	RefSeq	PHHY00000000	45	1	1	1	1	1
738	RefSeq	PHHZ00000000	45	1	1	1	1	1
980	RefSeq	PRDR00000000	45	1	1	1	1	1
208072207201-1	RefSeq	PEOJ00000000	45	1	1	1	1	1
209011300901-1	RefSeq	PEOS00000000	45	1	1	1	1	1
209013002001-1	RefSeq	PEOT00000000	45	1	1	1	1	1
209100702102-1	RefSeq	PEPC00000000	45	1	1	1	1	1
210050305001-1	RefSeq	PEPE00000000	45	1	1	1	1	1
213021206001-3	RefSeq	PEPW00000000	45	1	1	1	1	1
213123104301-4	RefSeq	PEQE00000000	45	1	1	1	1	1
12-4975*	SRA	SRR10076052	64	0	ND	ND	ND	ND
12-45328	SRA	SRR10076053	64	0	ND	ND	ND	ND
16-B20641	SRA	SRR10076071	64	0	ND	ND	ND	ND
SPSE-17-VL-OH-IN-PU-0016	SRA	SRR6160719	64	1	1	1	1	1
SPSE-17-VL-LA-KS-0039	SRA	SRR7239977	64	1	1	1	1	1
SPSE-18-VL-LA-TX-0016	SRA	SRR8176568	64	1	1	1	1	1
SPSE-18-VL-SD-OK-0017	SRA	SRR8236075	64	1	1	1	1	1
SPSE-18-VL-LA-ND-0031	SRA	SRR8815925	64	1	1	1	1	1
SPSE-18-VL-SD-OK-0026	SRA	SRR8815926	64	1	1	1	1	1
SPSE-18-VL-SD-OK-0042	SRA	SRR9028898	64	1	1	1	1	1
283734,1319	PATRIC	283734,1319	64	1	1	1	1	1
283734,1362	PATRIC	283734,1362	64	1	1	1	1	1
283734,1368	PATRIC	283734,1368	64	1	1	1	1	1
SP102a	RefSeq	QHIL00000000	64	1	1	1	1	1
SP18a	RefSeq	QHJC00000000	64	1	1	1	1	1
200	RefSeq	PHHV00000000	64	1	1	1	1	1
ST64-4	RefSeq	QQPM00000000	64	1	1	1	1	1
ST64-5	RefSeq	QQPN00000000	64	1	1	1	1	1
ST64-6	RefSeq	QQPO00000000	64	1	1	1	1	1
MAD401	RefSeq	QGPK00000000	68	1	1	1	1	1
283734,1315	PATRIC	283734,1315	68	1	1	1	1	1

283734,1316	PATRIC	283734,1316	68	1	1	1	1	1
283734,133	PATRIC	283734,133	68	1	1	1	1	1
283734,1334	PATRIC	283734,1334	68	1	1	1	1	1
283734,1344	PATRIC	283734,1344	68	1	1	1	1	1
283734,135	PATRIC	283734,135	68	1	1	1	1	1
283734,1352	PATRIC	283734,1352	68	1	1	1	1	1
283734,1364	PATRIC	283734,1364	68	1	1	1	1	1
283734,1385	PATRIC	283734,1385	68	1	1	1	1	1
283734,1393	PATRIC	283734,1393	68	1	1	1	1	1
283734,1401	PATRIC	283734,1401	68	1	1	1	1	1
283734,1452	PATRIC	283734,1452	68	1	1	1	1	1
23929	SRA	ERR175868	68	1	1	1	1	1
63228	RefSeq	CP015626	68	1	1	1	1	1
45665	SRA	SRR8092759	71	1	1	1	1	1
44812	SRA	SRR8092760	71	1	0	1	1	1
44407	SRA	SRR8092761	71	1	1	1	1	1
42203	SRA	SRR8092762	71	1	1	1	1	1
35686	SRA	SRR8092763	71	1	1	1	1	1
35681	SRA	SRR8092764	71	1	1	1	1	1
32980	SRA	SRR8092765	71	1	1	1	1	1
32959	SRA	SRR8092766	71	1	1	1	1	1
14-B13747	SRA	SRR10076046	71	0	ND	ND	ND	ND
13-46333	SRA	SRR10076050	71	0	ND	ND	ND	ND
15-B29911	SRA	SRR10076072	71	0	ND	ND	ND	ND
15-B26562	SRA	SRR10076075	71	0	ND	ND	ND	ND
15-B28096	SRA	SRR10076076	71	0	ND	ND	ND	ND
18-B24733	SRA	SRR10076079	71	0	ND	ND	ND	ND
SPSE-18-VL-NY-GA-0010	SRA	SRR8247815	71	1	1	1	1	1
283734,132	PATRIC	283734,132	71	1	1	1	1	1
283734,1335	PATRIC	283734,1335	71	1	1	1	1	1
283734,1336	PATRIC	283734,1336	71	1	1	1	1	1
283734,1356	PATRIC	283734,1356	71	1	0	1	1	1
283734,1438	PATRIC	283734,1438	71	1	1	1	1	1
283734,1458	PATRIC	283734,1458	71	1	1	1	1	1
69876	SRA	ERR144842	71	1	1	1	1	1
HH15	SRA	ERR144844	71	1	1	1	1	1
GL119A	SRA	ERR294366	71	1	1	1	1	1
GL151A	SRA	ERR294367	71	1	1	1	1	1
E140	RefSeq	ANOI00000000	71	1	1	1	1	1
SP131a	RefSeq	QHIB00000000	71	1	1	1	1	1
SP128a	RefSeq	QHIE00000000	71	1	1	1	1	1
SP106a	RefSeq	QHII00000000	71	1	1	1	1	1
SP105a	RefSeq	QHIJ00000000	71	1	1	1	1	1
SP86a	RefSeq	QHIO00000000	71	1	1	1	1	1
SP85a	RefSeq	QHIP00000000	71	1	1	1	1	1
SP35a	RefSeq	QHIT00000000	71	1	1	1	1	1
SP33a	RefSeq	QHIV00000000	71	1	1	1	1	1
SP29a	RefSeq	QHIZ00000000	71	1	1	1	1	1
SP28a	RefSeq	QHJA00000000	71	1	1	1	1	1
SP27a	RefSeq	QHJB00000000	71	1	1	1	1	1
81661 (20081661)	RefSeq	CP016073	71	1	1	1	0	1
335	RefSeq	PHHW00000000	71	1	1	1	1	1
473	RefSeq	PHHX00000000	71	1	1	1	1	1
651	RefSeq	PHIB00000000	71	1	1	1	1	1
586	RefSeq	PHIC00000000	71	1	1	1	1	1
476	RefSeq	PHID00000000	71	1	1	1	1	1

208071704702-1	RefSeq	PEOH00000000	71	1	1	1	1	1
208071804401-1	RefSeq	PEOI00000000	71	1	1	1	1	1
208073001801-1	RefSeq	PEOK00000000	71	1	1	1	1	1
208081905001-1	RefSeq	PEOL00000000	71	1	1	1	1	1
208082803802-1	RefSeq	PEON00000000	71	1	1	1	1	1
208090903101-3	RefSeq	PEOP00000000	71	1	1	1	1	1
208112602301-1	RefSeq	PEOQ00000000	71	1	1	1	1	1
209011202701-2	RefSeq	PEOR00000000	71	1	1	1	1	1
209020401401-1	RefSeq	PEOU00000000	71	1	1	1	1	1
209022503501-1	RefSeq	PEOV00000000	71	1	1	1	1	1
209031201604-5	RefSeq	PEOW00000000	71	1	1	1	1	1
209032500801-3	RefSeq	PEOX00000000	71	1	1	1	1	1
209040302601-1	RefSeq	PEOY00000000	71	1	1	1	1	1
209042702101-1	RefSeq	PEOZ00000000	71	1	1	1	1	1
209052604401-1	RefSeq	PEPA00000000	71	1	1	1	1	1
210062301601-1	RefSeq	PEPF00000000	71	1	1	1	1	1
211012802302-1	RefSeq	PEPK00000000	71	1	1	1	1	1
211041505801-1	RefSeq	PEPL00000000	71	1	1	1	1	1
211083101901-1	RefSeq	PEPM00000000	71	1	1	1	1	1
212042703101-1	RefSeq	PEPP00000000	71	1	1	1	1	1
213010701401-1	RefSeq	PEPT00000000	71	1	1	1	1	1
213121103601-1	RefSeq	PEQD00000000	71	1	1	1	1	1
G3C4	RefSeq	CP032682	71	1	1	1	1	1
SVA_MRSP_25130	SRA	ERR758366	71	1	1	1	1	1
SVA_MRSP_25131	SRA	ERR758380	71	1	1	1	1	1
SVA_MRSP_25136	SRA	ERR758381	71	1	1	1	1	1
SVA_MRSP_32123	SRA	ERR758382	71	1	1	1	1	1
SVA_MRSP_32569	SRA	ERR758383	71	1	1	1	1	1
SVA_MRSP_33004	SRA	ERR758384	71	1	1	1	1	1
SVA_MRSP_33008	SRA	ERR758385	71	1	1	1	1	1
SVA_MRSP_45655	SRA	ERR758386	71	1	1	1	1	1
SVA_MRSP_AB525	SRA	ERR758387	71	1	1	1	1	1
SVA_MRSP_AB557	SRA	ERR758388	71	1	1	1	1	1
SVA_MRSP_AB559	SRA	ERR758389	71	1	1	1	1	1
SVA_MRSP_AB560	SRA	ERR758390	71	1	1	1	1	1
SVA_MRSP_AB567	SRA	ERR758391	71	1	1	1	1	1
SVA_MRSP_AB587	SRA	ERR758392	71	1	1	1	1	1
SVA_MRSP_AB588	SRA	ERR758393	71	1	1	1	1	1
SVA_MRSP_AB616	SRA	ERR758394	71	1	1	1	1	1
SVA_MRSP_AB617	SRA	ERR758395	71	1	1	1	1	1
SVA_MRSP_AB619	SRA	ERR758396	71	1	1	1	1	1
SVA_MRSP_AB626	SRA	ERR758397	71	1	1	1	1	1
SVA_MRSP_AB728	SRA	ERR758398	71	1	1	1	1	1
SVA_MRSP_AB731	SRA	ERR758399	71	1	1	1	1	1
SVA_MRSP_AB766	SRA	ERR758401	71	1	1	1	1	1
SVA_MRSP_AB769	SRA	ERR758402	71	1	1	1	1	1
SVA_MRSP_AB789	SRA	ERR758403	71	1	1	1	1	1
283734,1317	PATRIC	283734,1317	84	1	1	1	1	1
283734,1318	PATRIC	283734,1318	84	1	1	1	1	1
283734,1338	PATRIC	283734,1338	84	1	1	1	1	1
283734,1366	PATRIC	283734,1366	84	1	1	1	1	1
SP111a	RefSeq	QHIH00000000	84	1	1	1	1	1
SP87a	RefSeq	QHIN00000000	84	1	1	1	1	1
SP83a	RefSeq	QHIR00000000	84	1	1	1	1	1
NA45 (UWMNA45)	RefSeq	CP016072	84	1	1	1	1	1
684	RefSeq	PHIA00000000	84	1	1	1	1	1

SPSE-18-VL-OH-WA-0002	SRA	SRR8062306	96	1	1	1	1	1
SPSE-18-VL-LA-KS-0016	SRA	SRR8176787	97	1	1	1	1	1
SPSE-18-VL-NY-GA-0015	SRA	SRR8360302	121	1	1	1	1	1
SL/154	RefSeq	MQNF00000000	121	1	1	1	1	1
15-3089	SRA	SRR10076032	150	0	ND	ND	ND	ND
15-3353	SRA	SRR10076074	150	0	ND	ND	ND	ND
SPSE-17-VL-NY-GA-0003	SRA	SRR6424825	150	1	1	1	1	1
SPSE-18-VL-NY-FL-0025	SRA	SRR8899776	150	1	1	1	1	1
283734,1346	PATRIC	283734,1346	150	1	1	1	1	1
283734,1349	PATRIC	283734,1349	150	1	0	1	1	1
283734,1372	PATRIC	283734,1372	150	1	1	1	1	1
SPSE-18-VL-LA-ND-0001	SRA	SRR12362133	155	1	1	1	1	1
SPSE-18-VL-SD-MI-0001	SRA	SRR7788256	155	1	1	1	1	1
SPSE-18-VL-NY-MS-0001	SRA	SRR8146147	155	1	1	1	1	1
SPSE-18-VL-LA-PA-Ryan-0013	SRA	SRR8176632	155	1	1	1	1	1
SPSE-18-VL-NY-MS-0016	SRA	SRR8360143	155	1	1	1	1	1
SPSE-18-VL-LA-PA-Ryan-0027	SRA	SRR8815909	155	1	1	1	1	1
SPSE-18-VL-LA-PA-Ryan-0031	SRA	SRR8815923	155	1	1	1	1	1
SPSE-18-VL-NY-GA-0019	SRA	SRR8864252	155	1	1	1	1	1
SPSE-18-VL-NY-MS-0043	SRA	SRR9016902	155	1	1	1	1	1
283734,1415	PATRIC	283734,1415	155	1	1	1	1	1
283734,1462	PATRIC	283734,1462	155	1	1	1	1	1
283734,1468	PATRIC	283734,1468	155	1	1	1	1	1
16-B22169	SRA	SRR10076067	157	0	ND	ND	ND	ND
SPSE-18-VL-NY-GA-0021	SRA	SRR8864266	157	1	1	1	1	1
SPSE-18-VL-SD-NC-0041	SRA	SRR9029455	157	1	1	1	1	1
283734,1331	PATRIC	283734,1331	169	1	1	1	1	1
15-36710	SRA	SRR10076031	181	0	ND	ND	ND	ND
18-H45776B	SRA	SRR10076045	181	0	ND	ND	ND	ND
14-43408	SRA	SRR10076048	181	0	ND	ND	ND	ND
16-34638	SRA	SRR10076063	181	0	ND	ND	ND	ND
16-31973	SRA	SRR10076066	181	0	ND	ND	ND	ND
18-H45776A	SRA	SRR10076068	181	0	ND	ND	ND	ND
16-31387	SRA	SRR10076069	181	0	ND	ND	ND	ND
15-38732	SRA	SRR10076078	181	0	ND	ND	ND	ND
SPSE-18-VL-LA-TX-0004	SRA	SRR12362129	181	1	1	1	1	1
SPSE-18-VL-LA-KY-0004	SRA	SRR12362138	181	1	1	1	1	1
SPSE-17-VL-LA-KY-0008	SRA	SRR6214505	181	1	1	1	1	1
SPSE-17-VL-LA-U-Penn-0006	SRA	SRR6374204	181	1	1	1	1	1
SPSE-17-VL-NY-MS-003	SRA	SRR6424470	181	1	1	1	1	1
SPSE-17-VL-SD-MI-0025	SRA	SRR7079317	181	1	1	1	1	1
SPSE-18-VL-SD-OK-0001	SRA	SRR7788249	181	1	1	1	1	1
SPSE-18-VL-NY-MS-0012	SRA	SRR8146029	181	1	1	1	1	1
SPSE-18-VL-SD-OK-0024	SRA	SRR8236053	181	1	1	1	1	1
SPSE-18-VL-SD-NC-0022	SRA	SRR8236062	181	1	1	1	1	1
SPSE-18-VL-SD-OK-0023	SRA	SRR8236070	181	1	1	1	1	1
SPSE-18-VL-LA-KS-0027	SRA	SRR8632535	181	1	1	1	1	1
SPSE-18-VL-SD-NC-0028	SRA	SRR8815962	181	1	1	1	1	1
SPSE-18-VL-NY-GAT-0004	SRA	SRR8146152	188	1	1	1	1	1
SPSE-18-VL-SD-IA-0022	SRA	SRR8236177	188	1	1	1	1	1
SPSE-18-VL-SD-OK-0044	SRA	SRR9029846	188	1	1	1	1	1
12-41269	SRA	SRR10076054	192	1	1	1	1	1
SPSE-18-VL-NY-MS-0023	SRA	SRR8360136	192	1	1	1	1	1
SPSE-17-VL-SD-OK-0014	SRA	SRR6311929	239	1	1	1	1	1
SPSE-18-VL-OH-ON-0005	SRA	SRR8062180	241	1	1	1	1	1
SPSE-18-VL-LA-KY-0031	SRA	SRR8815900	241	1	1	1	1	1

SPSE-18-VL-NY-NJ-0003	SRA	SRR8451763	257	1	1	1	1	1
283734,1377	PATRIC	283734,1377	257	1	1	1	1	1
41415	SRA	SRR8092757	258	1	1	1	1	1
33587	SRA	SRR8092758	258	1	1	1	1	1
44395	SRA	SRR8092774	258	1	1	1	1	1
42843	SRA	SRR8092775	258	1	1	1	1	1
44666	SRA	SRR8092776	258	1	1	1	1	1
44440	SRA	SRR8092777	258	1	1	1	1	1
SP84a	RefSeq	QHIQ00000000	258	1	1	1	1	1
210081303901-1	RefSeq	PEPG00000000	258	1	1	1	1	1
210083003701-1	RefSeq	PEPH00000000	258	1	1	1	1	1
212061102701-1	RefSeq	PEPQ00000000	258	1	1	1	1	1
212122401201-1	RefSeq	PEPS00000000	258	1	1	1	1	1
213012202401-1	RefSeq	PEPU00000000	258	1	1	1	1	1
213101103401-1	RefSeq	PEQB00000000	258	1	1	1	1	1
BNG1	SRA	ERR144839	260	1	1	1	1	1
14-35974	SRA	SRR10076047	261	0	ND	ND	ND	ND
SPSE-18-VL-OH-IN-0021	SRA	SRR8397379	261	1	1	1	1	1
1726	SRA	ERR144810	261	1	0	1	1	0
208090205001-1	RefSeq	PEOO00000000	261	1	1	1	1	1
209113002401-1	RefSeq	PEPD00000000	261	1	1	1	1	1
210101902302-1	RefSeq	PEPI00000000	261	1	1	1	1	1
213062502301-1	RefSeq	PEPZ00000000	261	1	1	1	1	1
208082101701-1	RefSeq	PEOM00000000	265	1	1	1	1	1
213101701201-2	RefSeq	PEQC00000000	265	1	1	1	1	1
SP34a	RefSeq	QHIU00000000	277	1	1	1	1	1
213032704301-1	RefSeq	PEPX00000000	277	1	1	1	1	1
SPSE-17-VL-OH-ON-0034	SRA	SRR6742563	282	1	1	1	0	1
SL/152	RefSeq	MQNE00000000	282	1	1	1	1	1
SL/164	RefSeq	MQNG00000000	282	1	1	1	1	1
MAD627	RefSeq	RJRN00000000	283	1	1	1	1	1
SPSE-17-VL-OH-WA-0003	SRA	SRR5928324	298	1	1	1	1	1
SPSE-18-VL-LA-KS-0023	SRA	SRR8176764	301	1	1	1	1	1
283734,1464	PATRIC	283734,1464	303	1	1	1	1	1
SPSE-18-VL-LA-PA-Ryan-0018	SRA	SRR8206962	307	1	1	1	1	1
212030604001-1	RefSeq	PEPN00000000	307	1	1	1	1	1
HKU10-03	RefSeq	NC_014925	308	1	1	1	1	1
SPSE-18-VL-SD-NC-0016	SRA	SRR8236040	311	1	1	1	1	1
213092504301-1	RefSeq	PEQA00000000	312	1	1	1	1	1
209080503802-1	RefSeq	PEPB00000000	334	1	1	1	1	1
210102003501-1	RefSeq	PEPJ00000000	336	1	1	1	1	1
213012301501-1	RefSeq	PEPV00000000	342	1	1	1	1	1
213041503101-1	RefSeq	PEPY00000000	346	1	1	1	1	1
212030802401-1	RefSeq	PEPO00000000	350	1	1	1	1	1
212112902001-1	RefSeq	PEPR00000000	350	1	1	1	1	1
SPSE-18-VL-NY-GA-0022	SRA	SRR9200333	373	1	1	1	1	1
SL/066	RefSeq	MQMO00000000	429	1	1	1	1	1
SPSE-17-VL-LA-ND-0004	SRA	SRR6374206	481	1	1	1	1	1
SPSE-18-VL-SD-MI-0037	SRA	SRR9028901	482	1	1	1	1	1
LMG 22219	RefSeq	MLGE00000000	494	1	1	1	1	1
44194	SRA	SRR8092767	496	1	1	1	1	1
44212	SRA	SRR8092768	496	1	1	1	1	1
44396	SRA	SRR8092769	496	1	1	1	1	1
42001	SRA	SRR8092770	496	1	1	1	1	1
41989	SRA	SRR8092771	496	1	1	1	1	1
42779	SRA	SRR8092772	496	1	1	1	1	1

42749	SRA	SRR8092773	496	1	1	1	1	1
43000	SRA	SRR8092778	496	1	1	1	1	1
42780	SRA	SRR8092779	496	1	1	1	1	1
SPSE-18-VL-LA-TX-0001	SRA	SRR7638094	496	0	ND	ND	ND	ND
SPSE-17-VL-LA-TX-0029	SRA	SRR6814849	496	1	1	1	1	1
SPSE-17-VL-LA-PA-Ryan-0030	SRA	SRR7239309	496	1	1	1	1	1
SPSE-18-VL-NY-NJ-0006	SRA	SRR8451759	496	1	1	1	1	1
SPSE-18-VL-LA-TX-0028	SRA	SRR8632711	496	1	1	1	1	1
SPSE-18-VL-SD-MI-0028	SRA	SRR8815957	496	1	1	1	1	1
SPSE-18-VL-SD-OK-0048	SRA	SRR9029852	496	1	1	1	1	1
SP132a	RefSeq	QHIA00000000	496	1	1	1	1	1
SP130a	RefSeq	QHIC00000000	496	1	1	1	1	1
SP32b	RefSeq	QHIW00000000	496	1	1	1	1	1
SP31a	RefSeq	QHIX00000000	496	1	1	1	1	1
SP30a	RefSeq	QHIY00000000	496	1	1	1	1	1
ST496-6	RefSeq	QQPJ00000000	496	1	1	1	1	1
283734,1369	PATRIC	283734,1369	497	1	1	1	1	1
SP103a	RefSeq	QHIK00000000	498	1	1	1	1	1
14-39020	SRA	SRR10076057	527	0	ND	ND	ND	ND
283734,1404	PATRIC	283734,1404	531	1	1	1	1	1
18-H48227	SRA	SRR10076036	551	0	ND	ND	ND	ND
SPSE-18-VL-LA-PA-Ryan-0021	SRA	SRR8206956	551	1	1	1	1	1
SPSE-18-VL-NY-GA-0020	SRA	SRR8864267	551	1	1	1	1	1
283734,1425	PATRIC	283734,1425	578	1	1	1	1	1
SPSE-18-VL-SD-MI-0031	SRA	SRR8815942	600	1	1	1	1	1
SPSE-18-VL-LA-KY-0019	SRA	SRR8632528	610	1	1	1	1	1
17-34192	SRA	SRR10076056	647	0	ND	ND	ND	ND
SPSE-18-VL-LA-PA-Ryan-0001	SRA	SRR7638090	705	1	1	1	1	1
283734,141	PATRIC	283734,141	718	1	1	1	1	1
17-31212	SRA	SRR10076062	749	0	ND	ND	ND	ND
16-33807	SRA	SRR10076065	749	0	ND	ND	ND	ND
SP127a	RefSeq	QHIF00000000	749	1	1	1	1	1
SP126a	RefSeq	QHIG00000000	749	1	1	1	1	1
283734,1327	PATRIC	283734,1327	752	1	0	1	1	1
283734,1325	PATRIC	283734,1325	754	1	1	1	1	1
283734,1341	PATRIC	283734,1341	755	1	1	1	1	1
283734,142	PATRIC	283734,142	755	1	1	1	1	1
SPSE-17-VL-SD-OK-0046	SRA	SRR7276444	757	1	1	1	1	1
SPSE-18-VL-NY-GA-0018	SRA	SRR8864015	757	1	1	1	1	1
283734,1354	PATRIC	283734,1354	758	1	1	1	1	1
283734,1394	PATRIC	283734,1394	759	1	1	1	1	1
283734,144	PATRIC	283734,144	760	1	1	1	1	1
17-23129	SRA	SRR10076058	762	1	1	1	1	1
283734,1454	PATRIC	283734,1454	762	1	1	1	1	1
283734,1445	PATRIC	283734,1445	767	1	1	1	1	1
283734,1429	PATRIC	283734,1429	768	1	1	1	1	1
283734,1323	PATRIC	283734,1323	769	1	1	1	1	1
283734,1397	PATRIC	283734,1397	771	1	1	1	1	1
283734,1378	PATRIC	283734,1378	801	1	1	1	1	1
283734,1384	PATRIC	283734,1384	802	1	1	1	1	1
283734,1409	PATRIC	283734,1409	804	1	1	1	1	1
283734,1423	PATRIC	283734,1423	804	1	1	1	1	1
283734,1326	PATRIC	283734,1326	806	1	1	1	1	1
283734,1314	PATRIC	283734,1314	808	1	1	1	1	1
283734,1414	PATRIC	283734,1414	811	1	1	1	1	1
283734,1434	PATRIC	283734,1434	811	1	1	1	1	1

283734,1455	PATRIC	283734,1455	812	1	1	1	1	1
46_57	RefSeq	SEZZ00000000	815	1	1	1	1	1
283734,145	PATRIC	283734,145	816	1	1	1	1	1
283734,1405	PATRIC	283734,1405	817	1	1	1	1	1
283734,1382	PATRIC	283734,1382	819	1	1	1	1	1
SPSE-18-VL-LA-KS-0006	SRA	SRR12362139	820	1	1	1	1	1
283734,1396	PATRIC	283734,1396	820	1	1	1	1	1
283734,1411	PATRIC	283734,1411	820	1	1	1	1	1
SPSE-18-VL-OH-WA-0026	SRA	SRR8945343	822	1	1	1	1	1
SPSE-18-VL-SD-IA-0040	SRA	SRR9029849	822	1	1	1	1	1
283734,1391	PATRIC	283734,1391	822	1	1	1	1	0
283734,1357	PATRIC	283734,1357	823	1	1	1	1	1
53_58	RefSeq	SEZX00000000	824	1	1	1	1	1
283734,1322	PATRIC	283734,1322	826	1	1	1	1	1
283734,1428	PATRIC	283734,1428	827	1	1	1	1	1
283734,1374	PATRIC	283734,1374	829	1	1	1	1	1
283734,1367	PATRIC	283734,1367	831	1	1	1	1	1
283734,1386	PATRIC	283734,1386	832	1	1	1	1	1
283734,1422	PATRIC	283734,1422	833	1	1	1	1	1
283734,1379	PATRIC	283734,1379	835	1	1	1	1	1
283734,14	PATRIC	283734,14	836	1	1	1	1	1
283734,1407	PATRIC	283734,1407	836	1	1	1	1	1
283734,136	PATRIC	283734,136	837	1	1	1	1	1
283734,1399	PATRIC	283734,1399	838	1	1	1	1	1
283734,1432	PATRIC	283734,1432	839	1	1	1	1	1
283734,138	PATRIC	283734,138	840	1	1	1	1	1
283734,1375	PATRIC	283734,1375	841	1	1	1	1	1
283734,1451	PATRIC	283734,1451	843	1	1	1	1	1
283734,1426	PATRIC	283734,1426	844	1	1	1	1	1
283734,1447	PATRIC	283734,1447	845	1	1	1	1	1
283734,1324	PATRIC	283734,1324	846	1	1	1	1	1
283734,1449	PATRIC	283734,1449	847	1	1	1	1	1
283734,1408	PATRIC	283734,1408	849	1	1	1	1	1
283734,143	PATRIC	283734,143	850	1	1	1	1	1
283734,1453	PATRIC	283734,1453	851	1	1	1	1	1
283734,1398	PATRIC	283734,1398	852	1	1	1	1	1
283734,1395	PATRIC	283734,1395	853	1	1	1	1	1
49_44	RefSeq	CP035743	856	1	1	1	1	1
283734,1406	PATRIC	283734,1406	858	1	1	1	1	1
283734,1446	PATRIC	283734,1446	859	1	1	1	1	1
283734,1365	PATRIC	283734,1365	861	1	1	1	1	1
283734,147	PATRIC	283734,147	862	1	1	1	1	1
283734,1392	PATRIC	283734,1392	863	1	1	1	1	1
51_92	RefSeq	CP035742	865	1	1	1	1	1
283734,1389	PATRIC	283734,1389	866	1	1	1	1	1
283734,1433	PATRIC	283734,1433	870	1	1	1	1	1
283734,139	PATRIC	283734,139	873	1	1	1	1	1
283734,1361	PATRIC	283734,1361	875	1	1	1	1	1
283734,1439	PATRIC	283734,1439	876	1	1	1	1	1
283734,1359	PATRIC	283734,1359	877	1	1	1	1	1
283734,1363	PATRIC	283734,1363	877	1	1	1	1	1
53_63	RefSeq	SEZW00000000	878	1	1	1	1	1
53_60	RefSeq	CP035741	879	1	1	1	1	1
283734,1353	PATRIC	283734,1353	880	1	1	1	1	1
283734,1417	PATRIC	283734,1417	883	1	1	1	1	1
283734,1436	PATRIC	283734,1436	884	1	1	1	1	1

113N	RefSeq	PJUR00000000	888	1	1	1	1	1
114N	RefSeq	PJUQ00000000	889	1	1	1	1	1
283734,1313	PATRIC	283734,1313	891	1	1	1	1	1
283734,1321	PATRIC	283734,1321	892	1	1	1	1	1
283734,1328	PATRIC	283734,1328	893	1	1	1	1	1
283734,1329	PATRIC	283734,1329	894	1	1	1	1	1
283734,1337	PATRIC	283734,1337	895	1	1	1	1	1
283734,1343	PATRIC	283734,1343	896	1	1	1	1	1
283734,1347	PATRIC	283734,1347	897	1	1	1	1	1
283734,1351	PATRIC	283734,1351	898	1	1	1	1	1
283734,1355	PATRIC	283734,1355	899	1	1	1	1	1
283734,137	PATRIC	283734,137	900	1	1	1	1	1
283734,1371	PATRIC	283734,1371	901	1	1	1	1	1
283734,1373	PATRIC	283734,1373	902	1	1	1	1	1
283734,1381	PATRIC	283734,1381	903	1	1	1	1	1
283734,1388	PATRIC	283734,1388	904	1	1	1	1	1
283734,1402	PATRIC	283734,1402	905	1	1	1	1	1
283734,1403	PATRIC	283734,1403	906	1	1	1	1	1
283734,1416	PATRIC	283734,1416	908	1	1	1	1	1
283734,1418	PATRIC	283734,1418	909	1	1	1	1	1
283734,1431	PATRIC	283734,1431	910	1	1	1	1	1
283734,1435	PATRIC	283734,1435	911	1	1	1	1	1
SPSE-18-VL-NY-GA-0011	SRA	SRR8360300	912	1	1	1	1	1
SPSE-17-VL-LA-U-Penn-0019	SRA	SRR6814921	916	1	1	1	1	1
SPSE-18-VL-SD-OK-0025	SRA	SRR8815940	917	1	1	1	1	1
283734,1469	PATRIC	283734,1469	917	1	1	1	1	1
18-B27335 (Abscess)	SRA	SRR10076080	923	0	ND	ND	ND	ND
SPSE-17-VL-LA-ND-0022	SRA	SRR6814918	923	1	1	1	1	1
SPSE-18-VL-SD-MI-0017	SRA	SRR8236043	923	1	1	1	1	1
SPSE-18-VL-NY-MS-0019	SRA	SRR8360135	923	1	1	1	1	1
SPSE-18-VL-SD-IA-0029	SRA	SRR8815904	923	1	1	1	1	1
SPSE-18-VL-LA-ND-0033	SRA	SRR8815917	923	1	1	1	1	1
SPSE-18-VL-SD-IA-0038	SRA	SRR9029553	923	1	1	1	1	1
283734,1427	PATRIC	283734,1427	923	1	1	1	1	1
424	RefSeq	PRDQ00000000	924	1	1	1	1	1
1019	RefSeq	PRDP00000000	930	1	1	1	1	1
SPSE-18-VL-LA-KS-0019	SRA	SRR8207014	978	1	1	1	1	1
9841998-1	RefSeq	JTKP00000000	990	1	1	1	1	1
9841787-1	RefSeq	JTKQ00000000	990	1	1	1	1	1
53_88	RefSeq	CP035740	1045	1	1	1	1	1
15-B18384	SRA	SRR10076034	1049	0	ND	ND	ND	ND
18-34619	SRA	SRR10076037	1049	0	ND	ND	ND	ND
17-B22336	SRA	SRR10076061	1049	0	ND	ND	ND	ND
16-B25196	SRA	SRR10076064	1049	0	ND	ND	ND	ND
15-B28539	SRA	SRR10076077	1049	0	ND	ND	ND	ND
18-34618	SRA	SRR10076038	1049	1	1	1	1	1
SPSE-18-VL-OH-IN-0003	SRA	SRR8062145	1049	1	1	1	1	1
SPSE-18-VL-SD-NC-0029	SRA	SRR8815921	1049	1	1	1	1	1
MAD568	RefSeq	QGPO00000000	1050	1	1	1	1	1
SPSE-18-VL-SD-OK-0003	SRA	SRR7788258	1055	1	1	1	1	1
SPSE-18-VL-LA-PA-Ryan-0006	SRA	SRR7765206	1060	1	1	1	1	1
SPSE-17-VL-OH-WA-0022	SRA	SRR6742584	1088	1	0	1	1	1
SP129a	RefSeq	QHID00000000	1171	1	1	1	1	1
SP53a	RefSeq	QHIS00000000	1172	1	1	1	1	1
SP101a	RefSeq	QHIM00000000	1173	1	1	1	1	1
SPSE-17-VL-NY-MS-0016	SRA	SRR6118665	1176	1	1	1	1	1

SPSE-17-VL-OH-WA-0013	SRA	SRR6160722	1177	1	1	1	1	1
SPSE-17-VL-LA-ND-0002	SRA	SRR6214465	1178	1	1	1	1	1
SPSE-17-VL-SD-OK-0019	SRA	SRR6312205	1179	1	1	1	1	1
SPSE-17-VL-NY-FL-0012	SRA	SRR6424306	1180	1	1	1	1	1
SPSE-17-VL-NY-MS-0031	SRA	SRR6795890	1181	1	1	1	1	1
SPSE-17-VL-OH-IN-PU-0006	SRA	SRR5928603	1211	1	1	1	1	1
SPSE-17-VL-NY-GA-0011	SRA	SRR6118626	1212	1	1	1	1	1
SPSE-17-VL-NY-FL-0013	SRA	SRR6118627	1213	1	1	1	1	1
SPSE-17-VL-OH-ON-0013	SRA	SRR6160723	1215	1	1	1	1	1
SPSE-18-VL-OH-GA-0002	SRA	SRR8062147	1215	1	1	1	1	1
SPSE-17-VL-LA-TX-0011	SRA	SRR6214501	1216	1	1	1	1	1
SPSE-17-VL-SD-NC-0010	SRA	SRR6311178	1217	1	1	1	1	1
SPSE-17-VL-SD-IA-0018	SRA	SRR6312081	1218	0	ND	ND	ND	ND
SPSE-17-VL-SD-MI-0020	SRA	SRR6312082	1219	1	1	1	1	1
SPSE-17-VL-SD-NC-0020	SRA	SRR6312088	1220	1	1	1	1	1
SPSE-17-VL-SD-MI-0001	SRA	SRR6312212	1221	1	1	1	1	1
SPSE-17-VL-SD-IA-0004	SRA	SRR6312289	1222	1	1	1	1	1
SPSE-17-VL-LA-TX-0023	SRA	SRR6374214	1223	1	1	1	1	1
SPSE-17-VL-OH-IN-PU-0034	SRA	SRR6742561	1224	1	1	1	1	1
SPSE-17-VL-OH-GA-0036	SRA	SRR6742574	1225	1	1	1	1	1
SPSE-17-VL-NY-NJ-0006	SRA	SRR6795845	1226	1	1	1	1	0
SPSE-17-VL-NY-FL-0015	SRA	SRR6795874	1227	1	1	1	1	1
SPSE-17-VL-NY-GA-0023	SRA	SRR6795894	1228	1	1	1	1	1
SPSE-17-VL-LA-KY-0033	SRA	SRR6814922	1229	1	1	1	1	1
SPSE-18-VL-LA-KS-0013	SRA	SRR8206947	1229	1	1	1	1	1
SPSE-18-VL-LA-KY-0022	SRA	SRR8815932	1229	1	1	1	1	1
ME4692	RefSeq	RJQH00000000	1291	1	1	1	1	1
SPSE-18-VL-LA-PA-Ryan-0022	SRA	SRR8206984	1295	1	1	1	1	1
SPSE-17-VL-SD-NC-0027	SRA	SRR6978943	1296	1	1	1	1	1
SPSE-18-VL-SD-IA-0048	SRA	SRR9029555	1297	1	1	1	1	1
SPSE-18-VL-LA-KS-0026	SRA	SRR8632715	1308	1	1	1	1	1
50_21	RefSeq	SEZY00000000	1335	1	1	1	1	1
SPSE-18-VL-SD-OK-0043	SRA	SRR9029421	1390	1	1	1	1	1
SPSE-18-VL-SD-NC-0020	SRA	SRR8236046	1417	1	1	1	1	1
SPSE-18-VL-SD-IA-0002	SRA	SRR7788476	1430	1	1	1	1	1
16-31143	SRA	SRR10076070	1431	0	ND	ND	ND	ND
SPSE-18-VL-OH-GA-0001	SRA	SRR8062140	1431	1	1	1	1	1
SPSE-18-VL-SD-NC-0026	SRA	SRR8815941	1431	1	1	1	1	1
SPSE-18-VL-NY-FL-0037	SRA	SRR9017117	1470	1	1	1	1	1
SPSE-18-VL-SD-OK-0033	SRA	SRR8815960	1573	1	1	1	1	1
SPSE-18-VL-LA-ND-0019	SRA	SRR12362143	1597	1	1	1	1	1
SPSE-17-VL-SD-IA-0045	SRA	SRR7278688	1598	1	1	1	1	1
SP79	RefSeq	AP019372	Novel	1	1	1	1	1
15-3070	SRA	SRR10076035	Novel	0	ND	ND	ND	ND
17-33972	SRA	SRR10076039	Novel	0	ND	ND	ND	ND
18-33914	SRA	SRR10076040	Novel	0	ND	ND	ND	ND
18-33717	SRA	SRR10076041	Novel	0	ND	ND	ND	ND
18-33072	SRA	SRR10076042	Novel	0	ND	ND	ND	ND
18-30846	SRA	SRR10076043	Novel	0	ND	ND	ND	ND
14-43376	SRA	SRR10076049	Novel	0	ND	ND	ND	ND
13-43410	SRA	SRR10076051	Novel	0	ND	ND	ND	ND
17-31831	SRA	SRR10076060	Novel	0	ND	ND	ND	ND
15-40292	SRA	SRR10076073	Novel	0	ND	ND	ND	ND
15-31914	SRA	SRR10076033	Novel	1	1	1	1	1
18-35753	SRA	SRR10076044	Novel	1	1	1	1	1
SPSE-18-VL-LA-ND-0023	SRA	SRR8300731	Novel	0	ND	ND	ND	ND

SPSE-18-VL-NY-FL-0033	SRA	SRR8902132	Novel	0	ND	ND	ND	ND
SPSE-18-VL-LA-KS-0001	SRA	SRR12362131	Novel	1	1	1	1	1
SPSE-18-VL-LA-KY-0032	SRA	SRR12362136	Novel	1	1	1	1	1
SPSE-18-VL-LA-KY-0006	SRA	SRR12362137	Novel	1	1	1	1	1
SPSE-18-VL-LA-KS-0004	SRA	SRR12362140	Novel	1	1	1	1	1
SPSE-17-VL-LA-KS-0011	SRA	SRR6374040	Novel	1	1	1	1	1
SPSE-17-VL-LA-KS-0015	SRA	SRR6374203	Novel	1	1	1	1	1
SPSE-17-VL-SD-OK-0029	SRA	SRR6978941	Novel	1	1	1	1	1
SPSE-17-VL-NY-FL-0030	SRA	SRR7064617	Novel	1	1	1	1	1
SPSE-17-VL-NY-NJ-003	SRA	SRR7066578	Novel	1	1	1	1	1
SPSE-17-VL-NY-MS-0040	SRA	SRR7066596	Novel	1	1	1	1	1
SPSE-17-VL-LA-TX-0041	SRA	SRR7239275	Novel	1	1	1	1	1
SPSE-17-VL-LA-ND-0029	SRA	SRR7239515	Novel	1	1	1	1	1
SPSE-17-VL-LA-KY-0040	SRA	SRR7239527	Novel	1	1	1	1	1
SPSE-17-VL-SD-NC-0047	SRA	SRR7278682	Novel	1	1	1	1	1
SPSE-17-VL-SD-MI-00039	SRA	SRR7497112	Novel	1	1	1	1	1
SPSE-18-VL-LA-KY-0001	SRA	SRR7638103	Novel	1	1	1	1	1
SPSE-18-VL-LA-ND-0004	SRA	SRR7647826	Novel	1	1	1	1	1
SPSE-18-VL-LA-ND-0006	SRA	SRR7765190	Novel	1	1	1	1	1
SPSE-18-VL-SD-OK-0002	SRA	SRR7788221	Novel	1	1	1	1	1
SPSE-18-VL-SD-OK-0005	SRA	SRR7788270	Novel	1	1	1	1	1
SPSE-18-VL-SD-IA-0003	SRA	SRR7788472	Novel	1	1	1	1	1
SPSE-18-VL-SD-OK-0006	SRA	SRR7788473	Novel	1	0	1	1	1
SPSE-18-VL-SD-MI-0005	SRA	SRR7788475	Novel	1	1	1	1	1
SPSE-18-VL-SD-IA-0001	SRA	SRR7788477	Novel	1	1	1	1	1
SPSE-18-VL-SD-MI-0006	SRA	SRR7788478	Novel	1	1	1	1	1
SPSE-18-VL-SD-IA-0006	SRA	SRR7788482	Novel	1	1	1	1	1
SPSE-18-VL-OH-ON-0001	SRA	SRR8062142	Novel	1	1	1	1	1
SPSE-18-VL-OH-WA-0005	SRA	SRR8062143	Novel	1	1	1	1	1
SPSE-18-VL-OH-WA-0006	SRA	SRR8062156	Novel	1	1	1	1	1
SPSE-18-VL-OH-IN-0001	SRA	SRR8062157	Novel	1	1	1	1	1
SPSE-18-VL-OH-ON-0003	SRA	SRR8062280	Novel	1	1	1	1	1
SPSE-18-VL-OH-GA-0006	SRA	SRR8062300	Novel	1	1	1	1	1
SPSE-18-VL-OH-IN-0006	SRA	SRR8062312	Novel	1	1	1	1	1
SPSE-18-VL-OH-GA-0014	SRA	SRR8144692	Novel	1	1	1	1	1
SPSE-18-VL-OH-ON-0023	SRA	SRR8144721	Novel	1	1	1	1	1
SPSE-18-VL-OH-IN-0014	SRA	SRR8144724	Novel	1	1	1	1	1
SPSE-18-VL-OH-GA-0020	SRA	SRR8144793	Novel	1	1	1	1	1
SPSE-18-VL-OH-GA-0016	SRA	SRR8144825	Novel	1	1	1	1	1
SPSE-18-VL-OH-ON-0024	SRA	SRR8144826	Novel	1	1	1	1	1
SPSE-18-VL-OH-GA-0021	SRA	SRR8144833	Novel	1	1	1	1	1
SPSE-18-VL-NY-MS-0004	SRA	SRR8146028	Novel	1	1	1	1	1
SPSE-18-VL-NY-GAT-0005	SRA	SRR8146136	Novel	1	1	1	1	1
SPSE-18-VL-NY-NJ-0001	SRA	SRR8146139	Novel	1	1	1	1	1
SPSE-18-VL-NY-MS-0006	SRA	SRR8146141	Novel	1	1	1	1	1
SPSE-18-VL-NY-NJ-0002	SRA	SRR8146143	Novel	1	1	1	1	1
SPSE-18-VL-NY-GAT-0007	SRA	SRR8146154	Novel	1	1	1	1	1
SPSE-18-VL-NY-FL-0006	SRA	SRR8146155	Novel	1	1	1	1	1
SPSE-18-VL-LA-PA-Ryan-0016	SRA	SRR8176616	Novel	1	1	1	1	1
SPSE-18-VL-LA-TX-0021	SRA	SRR8176618	Novel	1	1	1	1	1
SPSE-18-VL-LA-TX-0015	SRA	SRR8176636	Novel	1	1	1	1	1
SPSE-18-VL-LA-TX-0006	SRA	SRR8181816	Novel	1	1	1	1	1
SPSE-18-VL-LA-KY-0016	SRA	SRR8206965	Novel	1	1	1	1	1
SPSE-18-VL-LA-TX-0022	SRA	SRR8206993	Novel	1	1	1	1	1
SPSE-18-VL-LA-KS-0015	SRA	SRR8207004	Novel	1	1	1	1	1
SPSE-18-VL-LA-KY-0013	SRA	SRR8207006	Novel	1	1	1	1	1

SPSE-18-VL-LA-KY-0015	SRA	SRR8207020	Novel	1	1	1	1	1
SPSE-18-VL-SD-MI-0015	SRA	SRR8236044	Novel	1	1	1	1	1
SPSE-18-VL-SD-IA-0024	SRA	SRR8236047	Novel	1	1	1	1	1
SPSE-18-VL-SD-MI-0023	SRA	SRR8236048	Novel	1	1	1	1	1
SPSE-18-VL-SD-NC-0013	SRA	SRR8236049	Novel	1	1	1	1	1
SPSE-18-VL-SD-MI-0024	SRA	SRR8236050	Novel	1	1	1	1	1
SPSE-18-VL-SD-MI-0013	SRA	SRR8236054	Novel	1	1	1	1	1
SPSE-18-VL-SD-NC-0024	SRA	SRR8236059	Novel	1	1	1	1	1
SPSE-18-VL-SD-OK-0016	SRA	SRR8236061	Novel	1	1	1	1	1
SPSE-18-VL-SD-IA-0014	SRA	SRR8236063	Novel	1	1	1	1	1
SPSE-18-VL-SD-OK-0021	SRA	SRR8236064	Novel	1	1	1	1	1
SPSE-18-VL-SD-IA-0015	SRA	SRR8236072	Novel	1	1	1	1	1
SPSE-18-VL-NY-FL-0013	SRA	SRR8247809	Novel	1	1	1	1	1
SPSE-18-VL-NY-FL-0014	SRA	SRR8247817	Novel	1	1	1	1	1
SPSE-18-VL-NY-FL-0015	SRA	SRR8247819	Novel	1	1	1	1	1
SPSE-18-VL-LA-ND-0018	SRA	SRR8297600	Novel	1	1	1	1	1
SPSE-18-VL-LA-KY-0020	SRA	SRR8297632	Novel	1	1	1	1	1
SPSE-18-VL-LA-ND-0021	SRA	SRR8300733	Novel	1	1	1	1	1
SPSE-18-VL-LA-ND-0024	SRA	SRR8300738	Novel	1	1	1	1	1
SPSE-18-VL-NY-MS-0017	SRA	SRR8360152	Novel	1	1	1	1	1
SPSE-18-VL-NY-GA-0012	SRA	SRR8360303	Novel	1	1	1	1	1
SPSE-18-VL-OH-IN-0020	SRA	SRR8397396	Novel	1	1	1	1	1
SPSE-18-VL-OH-WA-0012	SRA	SRR8397438	Novel	1	1	1	1	1
SPSE-18-VL-OH-WA-0018	SRA	SRR8397607	Novel	1	1	1	1	1
SPSE-18-VL-NY-NJ-0007	SRA	SRR8451756	Novel	1	1	1	1	1
SPSE-18-VL-NY-NJ-0005	SRA	SRR8451757	Novel	1	1	1	1	1
SPSE-18-VL-NY-NJ-0004	SRA	SRR8451768	Novel	1	1	1	1	1
SPSE-18-VL-LA-TX-0033	SRA	SRR8632511	Novel	1	1	1	1	1
SPSE-18-VL-LA-TX-0035	SRA	SRR8632520	Novel	1	1	1	1	1
SPSE-18-VL-LA-TX-0036	SRA	SRR8632521	Novel	1	1	1	1	1
SPSE-18-VL-LA-TX-0030	SRA	SRR8632527	Novel	1	1	1	1	1
SPSE-18-VL-LA-KY-0033	SRA	SRR8767334	Novel	1	1	1	1	1
SPSE-18-VL-LA-ND-0030	SRA	SRR8767339	Novel	1	1	1	1	1
SPSE-18-VL-SD-OK-0030	SRA	SRR8815905	Novel	1	1	1	1	1
SPSE-18-VL-LA-PA-Ryan-0029	SRA	SRR8815906	Novel	1	1	1	1	1
SPSE-18-VL-SD-IA-0026	SRA	SRR8815908	Novel	1	1	1	1	1
SPSE-18-VL-LA-PA-Ryan-0026	SRA	SRR8815913	Novel	1	1	1	1	1
SPSE-18-VL-SD-NC-0033	SRA	SRR8815924	Novel	1	1	1	1	1
SPSE-18-VL-SD-IA-0035	SRA	SRR8815929	Novel	1	1	1	1	1
SPSE-18-VL-SD-MI-0035	SRA	SRR8815944	Novel	1	1	1	1	1
SPSE-18-VL-SD-IA-0033	SRA	SRR8815946	Novel	1	1	1	1	1
SPSE-18-VL-SD-MI-0036	SRA	SRR8815961	Novel	1	1	1	1	1
SPSE-18-VL-SD-IA-0030	SRA	SRR8815966	Novel	1	1	1	1	1
SPSE-18-VL-NY-GA-0016	SRA	SRR8864249	Novel	1	1	1	1	1
SPSE-18-VL-NY-MS-0024	SRA	SRR8864867	Novel	1	1	1	1	1
SPSE-18-VL-NY-FL-0019	SRA	SRR8864964	Novel	1	1	1	1	1
SPSE-18-VL-OH-ON-0030	SRA	SRR8868853	Novel	1	1	1	1	1
SPSE-18-VL-NY-FL-0022	SRA	SRR8895775	Novel	1	1	1	1	1
SPSE-18-VL-NY-FL-0026	SRA	SRR8899756	Novel	1	1	1	1	1
SPSE-18-VL-NY-FL-0029	SRA	SRR8899958	Novel	1	1	1	1	1
SPSE-18-VL-NY-MS-0026	SRA	SRR8902354	Novel	1	1	1	1	1
SPSE-18-VL-NY-MS-0029	SRA	SRR8923111	Novel	1	1	1	1	1
SPSE-18-VL-NY-MS-0034	SRA	SRR8923156	Novel	1	1	1	1	1
SPSE-18-VL-OH-WA-0024	SRA	SRR8945622	Novel	1	1	1	1	1
SPSE-18-VL-OH-WA-0025	SRA	SRR8945623	Novel	1	1	1	1	1
SPSE-18-VL-OH-WA-0022	SRA	SRR8945624	Novel	1	1	1	1	1

SPSE-18-VL-NY-MS-0045	SRA	SRR9016883	Novel	1	1	1	1	1
SPSE-18-VL-NY-MS-0042	SRA	SRR9016888	Novel	1	1	1	1	1
SPSE-18-VL-NY-MS-0040	SRA	SRR9016905	Novel	1	1	1	1	1
SPSE-18-VL-NY-MS-0048	SRA	SRR9017107	Novel	1	1	1	1	1
SPSE-18-VL-NY-FL-0036	SRA	SRR9017115	Novel	1	1	1	1	1
SPSE-18-VL-NY-FL-0046	SRA	SRR9017126	Novel	1	1	1	1	1
SPSE-18-VL-NY-FL-0045	SRA	SRR9017130	Novel	1	1	1	1	1
SPSE-18-VL-SD-MI-0039	SRA	SRR9028900	Novel	1	1	1	1	1
SPSE-18-VL-SD-NC-0044	SRA	SRR9029416	Novel	1	1	1	1	1
SPSE-18-VL-SD-MI-0047	SRA	SRR9029422	Novel	1	1	1	1	1
SPSE-18-VL-SD-NC-0039	SRA	SRR9029432	Novel	1	1	1	1	1
SPSE-18-VL-SD-NC-0038	SRA	SRR9029450	Novel	1	1	1	1	1
SPSE-18-VL-SD-IA-0037	SRA	SRR9029451	Novel	1	1	1	1	1
SPSE-18-VL-SD-IA-0039	SRA	SRR9029543	Novel	1	1	1	1	1
SPSE-18-VL-SD-MI-0046	SRA	SRR9029548	Novel	1	1	1	1	1
SPSE-18-VL-NY-GA-0024	SRA	SRR9200080	Novel	1	1	1	1	1
SPSE-18-VL-NY-GA-0025	SRA	SRR9200108	Novel	1	1	1	1	1
SPSE-18-VL-NY-GA-0023	SRA	SRR9200707	Novel	1	1	1	1	1

Supplemental references

1. Monk IR, Shah IM, Xu M, Tan MW, Foster TJ. Transforming the untransformable: application of direct transformation to manipulate genetically *Staphylococcus aureus* and *Staphylococcus epidermidis*. mBio. 2012;3(2). doi: 10.1128/mBio.00277-11. PubMed PMID: 22434850; PubMed Central PMCID: PMC3312211.
2. Savini V, Barbarini D, Polakowska K, Gherardi G, Bialecka A, Kasprowicz A, et al. Methicillin-resistant *Staphylococcus pseudintermedius* infection in a bone marrow transplant recipient. Journal of clinical microbiology. 2013;51(5):1636-8. doi: 10.1128/JCM.03310-12. PubMed PMID: 23486715; PubMed Central PMCID: PMC3647946.
3. Bannoehr J, Ben Zakour NL, Waller AS, Guardabassi L, Thoday KL, van den Broek AH, et al. Population genetic structure of the *Staphylococcus intermedius* group: insights into *agr* diversification and the emergence of methicillin-resistant strains. Journal of bacteriology. 2007;189(23):8685-92. doi: 10.1128/JB.01150-07. PubMed PMID: 17905991; PubMed Central PMCID: PMC2168937.
4. Solyman SM, Black CC, Duim B, Perreten V, van Duijkeren E, Wagenaar JA, et al. Multilocus sequence typing for characterization of *Staphylococcus pseudintermedius*. Journal of clinical microbiology. 2013;51(1):306-10. doi: 10.1128/JCM.02421-12. PubMed PMID: 23115265; PubMed Central PMCID: PMC3536184.
